# Evaluation of a multi-component AI-guided mechanistic reasoning practical in experimental pharmacology: a single-group pre-post study

**DOI:** 10.3389/fphar.2026.1937176

**Published:** 2026-09-21

**Authors:** Maan H. Harbi

**Affiliations:** Pharmacology and Toxicology Department, College of Pharmacy, Umm Al-Qura University, Makkah, Saudi Arabia

**Keywords:** antithrombotic pharmacology, artificial intelligence, experimental pharmacology, mechanistic reasoning, pharmacology education, pharmacy education

## Abstract

Background. Experimental pharmacology requires students to interpret laboratory findings, identify affected physiological pathways, and infer underlying drug mechanisms. While artificial intelligence (AI) is increasingly explored in health professions education, concerns remain regarding passive learning and overreliance on generated answers. This study evaluated the feasibility, acceptability, and short-term outcomes of a structured, multi-component practical designed to support active reasoning in antithrombotic pharmacology. Methods. A single-group pre-post educational intervention was conducted among 44 second-year PharmD students. The intervention included a baseline assessment, an experimental logic mini-brief, an AI-supported mechanistic reasoning practical using a standardized reasoning prompt, an independent post-test, and a perception survey. The primary outcome was total assessment score change. Secondary outcomes included domain-specific scores, performance during the AI-supported practical, confidence, perceived AI helpfulness, student perceptions, and associations between practical performance and learning outcomes. Results. Total assessment scores improved significantly from 76.82% ± 25.06% at baseline to 85.80% ± 21.27% after the intervention, with a mean gain of 8.98 ± 20.53 percentage points (Wilcoxon signed-rank test, Z = −2.597, p = 0.009, r = 0.39). Significant pre-post improvements were observed in coagulation pharmacology (p = 0.001, r = 0.49) and a smaller uncorrected improvement occurred in platelet pharmacology (p = 0.048, r = 0.30), while experimental reasoning scores increased numerically but did not reach statistical significance (p = 0.219, r = 0.19). Among AI-supported practical completers (n = 43), the mean Step 3 reasoning score was 17.88 ± 4.01 out of 21 (85.1% ± 19.1%). Accuracy was highest for experimental interpretation (87.0%), followed by mechanism or pathway identification (85.0%) and drug-class identification (83.4%). Step 3 performance was positively associated with post-test score (Spearman’s rho = 0.606, p < 0.001). The core perception and acceptability items showed excellent internal consistency (Cronbach’s alpha = 0.935), and student perceptions were generally favorable. Conclusion. A multi-component pharmacology practical containing an AI-guided reasoning prompt was feasible, acceptable, and associated with improved short-term assessment scores. Given the single-group pre-post design, we cannot isolate the specific AI contribution from co-occurring instructional elements, such as the mini-brief, case-based practice, and retrieval-practice testing effects. Controlled studies are needed to assess causal efficacy and long-term retention.

## Introduction

Experimental pharmacology is a fundamental component of pharmacy education because it helps students understand how drug effects are investigated, interpreted, and translated into clinical practice. Unlike traditional knowledge-based learning, experimental pharmacology requires learners to interpret laboratory findings, identify affected physiological pathways, and infer underlying pharmacological mechanisms. Developing these skills is essential for building mechanistic understanding and supporting evidence-based decision-making in future clinical practice.

Students often find experimental pharmacology challenging because successful interpretation of experimental data requires integration of concepts from physiology, pharmacology, and pathophysiology (Masava et al., 2023). Tasks such as interpreting platelet aggregation curves, coagulation assays, and drug-response patterns demand higher-order reasoning rather than simple recall of factual knowledge (Masava et al., 2023; Patino et al., 2024). Consequently, there is increasing interest in educational strategies that can support students in developing these reasoning skills.

Artificial intelligence (AI) in higher education is being used across several instructional functions, including intelligent tutoring, adaptive support, assessment, and prediction, but the evidence base remains uneven and much of the literature is still early-stage rather than strongly causal or outcome-focused (Bates et al., 2020; Crompton and Burke, 2023). In health professions education, early large-language-model (LLM) studies accelerated interest in AI as a possible educational aid or virtual tutor, while also requiring careful evaluation and human oversight (Gilson et al., 2023; Kung et al., 2023).

Recent advances in AI, particularly LLMs such as ChatGPT, Claude, and Gemini, have drawn attention within health professions education (Preiksaitis and Rose, 2023; Patino et al., 2024). These tools may support learning through interactive explanations, question answering, and personalized feedback. Several studies have reported positive student perceptions of AI-assisted learning and suggested benefits for knowledge acquisition and self-directed learning (Preiksaitis and Rose, 2023; Sridharan and Sequeira, 2024). As a result, AI is increasingly being explored as a supplementary educational resource in medicine, pharmacy, and other healthcare disciplines (Patino et al., 2024; Feigerlova et al., 2025).

Despite these opportunities, concerns have been raised regarding the educational use of generative AI (Fan et al., 2024). When used without appropriate guidance, AI systems may encourage passive learning, cognitive offloading, and overreliance on generated responses (Darvishi et al., 2024; Fan et al., 2024). Students may obtain correct answers without engaging in the reasoning processes required for meaningful learning and knowledge transfer (Fan et al., 2024). Consequently, recent educational literature has emphasized that the effectiveness of AI depends not only on the technology itself but also on how it is integrated into instructional design (Feigerlova et al., 2025). Structured approaches that encourage questioning, reflection, and justification of reasoning may therefore be more educationally valuable than unrestricted AI use (Feigerlova et al., 2025).

Instructional scaffolding provides a useful framework for this type of learning task. Scaffolds provide temporary guidance during complex learning activities while requiring learners to generate and justify their own responses (Masava et al., 2023). In the present intervention, the standardized AI prompt was designed to provide this type of guidance by asking students to interpret the experimental readout, identify the affected physiological pathway, and infer the likely mechanism before selecting a drug class. This differs from unrestricted answer generation because the intended sequence preserves active student reasoning. Although AI has been studied increasingly in health professions education, relatively little work has examined constrained AI-guided support for data-to-mechanism reasoning in experimental pharmacology (Preiksaitis and Rose, 2023; Sridharan and Sequeira, 2024).

Within pharmacology education, relatively little research has examined the use of AI to support interpretation of experimental data and mechanistic reasoning. Most published studies have focused on knowledge retrieval, question answering, or student perceptions of AI technologies (Preiksaitis and Rose, 2023; Patino et al., 2024). There is a need for educational approaches that use AI to guide students through structured pharmacological reasoning while maintaining active engagement with experimental data.

We therefore developed a structured educational intervention combining an experimental logic mini-brief with an AI-guided mechanistic reasoning practical focused on antiplatelet and anticoagulant pharmacology. The intervention used an AI-guided reasoning prompt intended to guide students through a sequential process of interpreting experimental findings, identifying affected pathways, and inferring pharmacological mechanisms. Rather than being used as a direct answer source, the AI prompt was designed to support scaffolded reasoning, active learning, and mechanistic understanding.

This distinction is important for this study because guided retrieval and explanation are theoretically different from simply revealing answers or re-exposing learners to content: retrieval practice is itself a learning event and is associated with stronger long-term retention than restudy alone (Roediger and Karpicke, 2006; Roediger and Butler, 2011). For pharmacology teaching in particular, the target is not only factual recall but explanation-oriented reasoning about how interventions alter biological systems, making it useful to frame the intervention in relation to mechanistic disciplinary thinking rather than answer delivery alone (Talanquer, 2018).

The aim of this study was to evaluate short-term learning outcomes, practical performance, and student perceptions following a multi-component experimental pharmacology practical incorporating an AI-guided mechanistic reasoning activity. Specifically, the study assessed changes in pharmacology knowledge, performance during AI-supported reasoning activities, and student perceptions of the educational approach.

## Methods

### Study design and setting

This study employed a single-group pre-post educational intervention design to evaluate short-term learning outcomes and student perceptions following a multi-component experimental pharmacology practical that included an AI-guided mechanistic reasoning activity. The design was intended as an initial educational evaluation and was not designed to isolate the independent effect of AI. The intervention was implemented as part of a second-year PharmD pharmacology practical session focused on platelet function, antiplatelet drugs, coagulation pathways, and anticoagulant pharmacology. Participation involved completion of the educational activities and associated assessments.

The intervention consisted of five sequential stages: a baseline assessment, an experimental logic mini-brief, an AI-supported mechanistic reasoning practical, an independent post-intervention assessment, and a perception and acceptability survey. Figure 1 summarizes the study workflow and educational intervention. The educational materials and study instruments are provided as Supplementary Materials to support replication of the intervention. These include the baseline assessment, experimental logic mini-brief, AI-supported practical worksheet, post-intervention assessment, and perception and acceptability survey (Supplementary Materials S1–S5).

**FIGURE 1:**
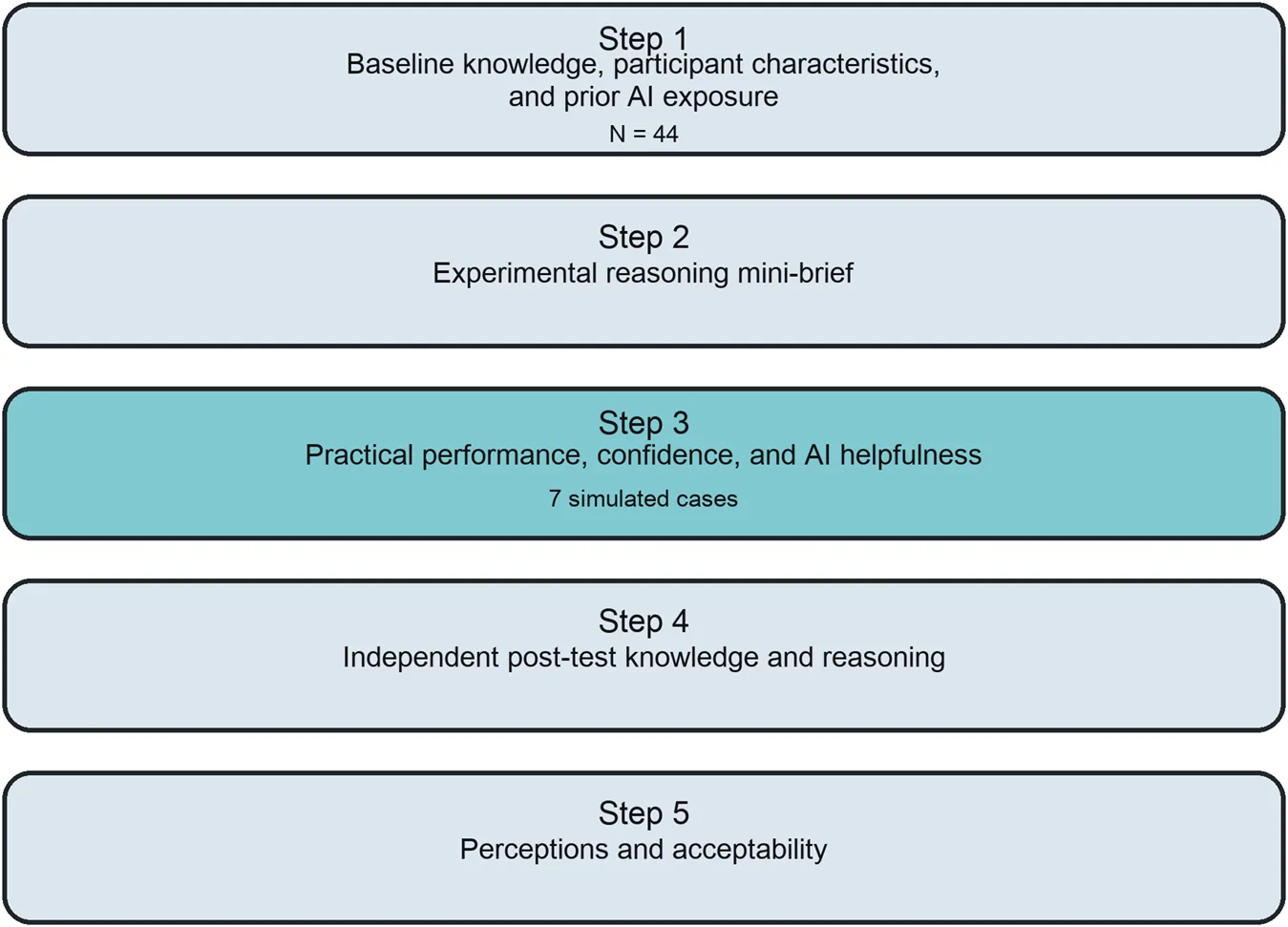
Study workflow, educational intervention, and measured outcomes. Participants (N = 44) completed a baseline assessment of pharmacology knowledge, participant characteristics, and prior AI exposure; received an experimental reasoning mini-brief; completed seven AI-supported simulated cases assessing practical performance, confidence, and perceived AI helpfulness; completed an independent post-test of knowledge and reasoning; and finally reported their perceptions and acceptability of the intervention.

### Educational intervention

#### Development of the AI prompt and instructional framework

The intervention was built around a structured reasoning sequence in which students moved from the experimental finding to the affected pathway, then to the likely mechanism and drug class. This sequence was chosen because the practical focused on data interpretation rather than recall of drug names. Students were expected to start with the laboratory or simulation finding, decide what physiological process was altered, and then infer the most likely pharmacological explanation.

A standard AI prompt was prepared before the session. The prompt instructed the AI system to act as a guided reasoning assistant rather than an answer provider. It asked the AI to guide students with questions, ask them to justify their interpretation, and keep the discussion focused on the experimental data. Students were told not to request direct answers from the AI system. The same prompt was used across the practical to keep the AI-supported activity consistent between students.

#### Step 1: baseline assessment

The baseline assessment measured students’ starting knowledge and reasoning before the AI-guided practical. It included 20 multiple-choice questions. Five questions assessed platelet pharmacology, five assessed coagulation pharmacology, and ten assessed experimental reasoning and data interpretation.

The questions covered platelet aggregation pathways, antiplatelet drug mechanisms, coagulation tests, PT and aPTT interpretation, and inference of drug mechanisms from experimental findings. The baseline form also collected age category, GPA category, prior exposure to antiplatelet and anticoagulant drugs, attendance at related cardiovascular pharmacology lectures, pharmacy work experience, confidence in interpreting pharmacology laboratory data, and previous use of AI tools for studying. The full baseline assessment instrument is provided in Supplementary Material S1.

#### Step 2: experimental logic mini-brief

Following the baseline assessment, students received a structured mini-brief introducing the experimental reasoning framework used throughout the practical activity. The mini-brief reviewed principles of platelet aggregation testing, interpretation of light transmission aggregometry findings, interpretation of PT and aPTT results, identification of affected physiological pathways, and mechanism-based drug inference. Students were introduced to a standardized reasoning sequence: **Experimental finding → affected pathway → mechanism → drug class**. The purpose of the mini-brief was to establish a common approach before engagement with the AI-supported practical activity. The mini-brief slides used to introduce the experimental logic of platelet aggregation, coagulation testing, and mechanism-based inference are provided in Supplementary Material S2.

#### Step 3: AI-supported experimental reasoning practical

Students then completed seven simulated experimental pharmacology cases. Three cases focused on platelet function and antiplatelet drugs, three focused on coagulation and anticoagulant drugs, and one combined platelet and coagulation findings. Each case presented simulated experimental data such as platelet aggregation findings, coagulation test results, or integrated pharmacological observations.

Each case included three scored reasoning questions: experimental interpretation, mechanism or pathway identification, and drug-class identification. Each correct answer was given one point, giving a total Step 3 reasoning score of 21 points across the seven cases. Each component score was also calculated across the seven cases, giving separate scores out of 7 for experimental interpretation, mechanism or pathway identification, and drug-class identification. These component scores were reported as raw scores and percentages.

Students also rated their confidence after each case using a five-point scale. Mean confidence was calculated across the seven cases. Students also reported whether AI helped their reasoning for each case. AI helpfulness was calculated as the percentage of cases in which each student answered yes.

#### AI system and prompt

Students completed the AI-supported practical individually using personal devices. The activity used commercially available generative AI tools accessed during May 2026. The exact platform, model version, subscription tier, personalization settings, and platform-level configuration used by each student were not independently logged or standardized. A standardized prompt was provided to all students to reduce variation in the intended instructional use of AI, but differences in model capability and prior student familiarity with AI may still have affected the interaction.

The prompt was designed to support reasoning rather than answer generation. The prompt directed the AI system to function as a guided reasoning assistant by asking guiding questions and encouraging students to identify the key experimental abnormality, the affected physiological pathway, the most likely pharmacological mechanism, and the most likely drug class. Students were explicitly instructed not to request direct answers from the AI system. The full AI-supported practical worksheet, including the approved AI prompt, simulated cases, structured reasoning questions, confidence items, and AI helpfulness items, is provided in Supplementary Material S3.

#### Step 4: post-intervention assessment

Immediately following completion of the AI-supported practical, students completed a post-intervention assessment. The post-test used a parallel 20-item form designed to match the baseline assessment in domain coverage and learning objectives. Both forms included five platelet pharmacology items, five coagulation pharmacology items, and ten experimental reasoning items.

The assessment was completed independently without AI assistance or external learning resources. The post-intervention assessment instrument is provided in Supplementary Material S4.

#### Step 5: perception and acceptability survey

After the post-test, students completed a perception and acceptability survey evaluating their experience with the AI-guided learning activity. The survey included 12 Likert-scale items assessing perceived learning benefit, confidence in interpreting experimental pharmacology data, ability to reason from experimental findings to drug mechanisms, perceived usefulness of AI guidance, acceptability of the educational approach, and support for future use of AI-guided reasoning activities in pharmacology practicals. Responses were coded on a five-point scale from 1 = strongly disagree to 5 = strongly agree. For item-level summaries, agreement was defined as a response of agree or strongly agree. The full perception and acceptability survey is provided in Supplementary Material S5.

### Outcome measures

The primary outcome was the change in overall pharmacology assessment score from baseline to post-intervention. Baseline and post-test total scores were expressed as percentages. Secondary outcomes included domain-specific changes in platelet pharmacology, coagulation pharmacology, and experimental reasoning scores; performance during the AI-supported practical; accuracy of experimental interpretation, mechanism/pathway identification, and drug-class identification during Step 3; mean confidence during the practical; perceived AI helpfulness; student perceptions of the intervention; and associations between practical performance, confidence, AI helpfulness, baseline score, post-test score, and learning gain. The content of each assessment and survey instrument is available in the Supplementary Materials.

### Data management and cleaning

Data from all study stages were exported into Microsoft Excel for cleaning and preparation before analysis in SPSS. Duplicate responses were identified using anonymous participant identifiers. Duplicate submissions occurred in a small number of participants across the baseline assessment, post-intervention assessment, and perception survey, but none occurred during the AI-supported practical. Students did not receive scores, correct answers, or corrective feedback after submission. For duplicate records, the final and most complete submission associated with each anonymous identifier was retained. Baseline and post-test assessment items were scored dichotomously as correct or incorrect, and total assessment scores were converted to percentages. Domain scores were calculated using their original scales, with platelet pharmacology and coagulation pharmacology each scored out of 5 and experimental reasoning scored out of 10. Learning gain was calculated as the post-test total percentage score minus the baseline total percentage score.

Missing responses were handled on an analysis-specific basis. Students were excluded only from analyses requiring the missing outcome and were retained in all analyses for which valid data were available. Accordingly, analyses of baseline characteristics, baseline assessment, post-test assessment, perception survey responses, and baseline-to-post-test learning outcomes included 44 students, whereas Step 3-specific practical performance analyses included 43 students.

### Statistical analysis

Statistical analyses were performed using IBM SPSS Statistics (Version 25; IBM Corp., Armonk, NY, United States). Descriptive statistics were used to summarize participant characteristics, assessment scores, practical performance, and survey responses. Continuous variables were reported as means and standard deviations or medians and interquartile ranges as appropriate. Categorical variables were reported as frequencies and percentages. Normality of score distributions was assessed using the Shapiro-Wilk test. Because assessment scores were not normally distributed, nonparametric analyses were used for pre-post comparisons.

Baseline and post-intervention total assessment scores were compared using the Wilcoxon signed-rank test. Domain-specific pre-post comparisons for platelet pharmacology, coagulation pharmacology, and experimental reasoning were also evaluated using Wilcoxon signed-rank tests. Domain-specific comparisons were considered exploratory. No adjustment for multiple comparisons was applied, and these results should therefore be interpreted cautiously. Effect sizes for Wilcoxon signed-rank tests were calculated as r = |Z|/√N, where Z is the standardized test statistic and N is the number of paired observations. Performance during the AI-supported practical was analyzed descriptively. The Step 3 total reasoning score was calculated from 21 scored items across seven simulated cases. Component scores for experimental interpretation, mechanism/pathway identification, and drug-class identification were calculated out of 7 and converted to percentages for graphical presentation. Confidence was summarized as the mean confidence score across the seven cases, and AI helpfulness was summarized as the percentage of cases in which each student reported that AI helped their reasoning.

Internal consistency reliability of the baseline assessment, post-test assessment, and perception survey was evaluated using Cronbach’s alpha. For the dichotomously scored baseline and post-test assessments, Cronbach’s alpha was interpreted as equivalent to Kuder–Richardson Formula 20 (KR-20). The 10 core perception and acceptability items were assessed for internal consistency using Cronbach’s alpha. The two safety/limitations items addressing AI-related confusion and the need for instructor guidance were analyzed separately at the item level because they represented distinct implementation considerations rather than positive acceptability. All survey items were additionally summarized using item-level descriptive statistics, including mean, standard deviation, median, interquartile range, and the proportion of students who agreed or strongly agreed with each item.

Associations between Step 3 practical performance, post-test achievement, baseline performance, learning gain, confidence, and AI helpfulness were evaluated using Spearman’s rank correlation coefficient. Step 3-related correlation analyses included only students who completed the AI-supported practical, whereas correlations involving baseline and post-test outcomes without Step 3 variables included all students with available paired data.

### Ethical considerations

This study was approved by the Biomedical Research Ethics Committee at Umm Al-Qura University, Approval No. HAPO-02-K-012-2026-05-3422, and was conducted in accordance with the Declaration of Helsinki. The educational activity was implemented as part of routine course delivery. Participant responses were anonymized using study identifiers before analysis. No personally identifiable information was collected, and all data were analyzed and reported in aggregate form.

## Results

### Participant characteristics

A total of 44 second-year PharmD students were included in the final analytical sample. Most participants were aged 36–40 years (25/44, 56.8%) or older than 40 years (17/44, 38.6%). The majority reported a GPA between 3.5 and 4.0 (27/44, 61.4%), while 4 students (9.1%) reported a GPA above 4.0. Most students had previously studied antiplatelet drugs (38/44, 86.4%) and anticoagulant drugs (39/44, 88.6%) in detail. In addition, 32 students (72.7%) reported attending the relevant cardiovascular pharmacology lectures, and 32 students (72.7%) reported previous hospital pharmacy work experience.

Regarding baseline confidence and AI exposure, 19 students (43.2%) reported being confident or very confident in interpreting pharmacology laboratory data. AI use for studying was common: 18 students (40.9%) reported using AI tools sometimes, while 17 students (38.7%) reported using them often or very often. Previous use of AI for pharmacology learning was reported by 32 students (72.7%), and previous use of AI to interpret data, graphs, or laboratory results was reported by 27 students (61.4%). Baseline characteristics are summarized in Table 1.

**TABLE 1:** Baseline characteristics of study participants (N = 44).

Characteristic	n (%)
Age category, years	
31–35	2 (4.5)
36–40	25 (56.8)
>40	17 (38.6)
Self-reported GPA category	
<2.5	1 (2.3)
2.5–2.99	3 (6.8)
3.0–3.49	9 (20.5)
3.5–4.0	27 (61.4)
>4.0	4 (9.1)
Previously studied antiplatelet drugs in detail	
No	3 (6.8)
Yes	38 (86.4)
Not sure	3 (6.8)
Previously studied anticoagulant drugs in detail	
No	2 (4.5)
Yes	39 (88.6)
Not sure	3 (6.8)
Attended relevant cardiovascular pharmacology lectures	
No	5 (11.4)
Yes	32 (72.7)
Partially	7 (15.9)
Previous pharmacy work experience	
No	3 (6.8)
Community pharmacy	5 (11.4)
Hospital pharmacy	32 (72.7)
Other healthcare setting	4 (9.1)
Confidence in interpreting pharmacology laboratory data	
Not confident	2 (4.5)
Slightly confident	12 (27.3)
Moderately confident	11 (25.0)
Confident	11 (25.0)
Very confident	8 (18.2)
Frequency of AI use for studying	
Never	6 (13.6)
Rarely	3 (6.8)
Sometimes	18 (40.9)
Often	9 (20.5)
Very often	8 (18.2)
Previous use of AI for pharmacology learning	
No	12 (27.3)
Yes	32 (72.7)
Previous use of AI to interpret data, graphs, or laboratory results	
No	17 (38.6)
Yes	27 (61.4)
Confidence using AI for academic learning	​
Slightly confident	9 (20.5)
Moderately confident	18 (40.9)
Confident	11 (25.0)
Very confident	6 (13.6)
Characteristic	n (%)

Percentages are based on valid responses. No missing responses were reported for the variables shown. Variables were collected during the Step 1 baseline assessment, including demographics, educational exposure, and AI, familiarity/confidence items.

The assessment instruments demonstrated excellent internal consistency reliability. The baseline assessment had a Cronbach’s alpha of 0.909 across 20 dichotomously scored items, while the post-test assessment had a Cronbach’s alpha of 0.914 across 20 items. Because these items were scored as correct or incorrect, Cronbach’s alpha is equivalent to KR-20.

### Overall pre- and post-intervention learning outcomes

Overall assessment scores improved following the intervention. The mean total assessment score increased from 76.82% ± 25.06% at baseline to 85.80% ± 21.27% after the intervention, corresponding to a mean gain of 8.98 ± 20.53 percentage points. Median scores increased from 85.00% (IQR 55.00–100.00) to 95.00% (IQR 80.00–100.00). The Wilcoxon signed-rank test showed that this improvement was statistically significant (Z = −2.597, p = 0.009), with a moderate effect size (r = 0.39). Twenty-one students improved, nine showed lower post-test scores, and 14 had unchanged scores. These findings are presented in Table 2 and visualized in Figure 2.

**TABLE 2:** Overall pre- and post-intervention assessment scores.

Outcome	Baseline	Post-test	Change	Test statistic	p-value
Total assessment score, mean ± SD (%)	76.82 ± 25.06	85.80 ± 21.27	+8.98 ± 20.53	Z = −2.597	0.009
Total assessment score, median (IQR) (%)	85.00 (55.00–100.00)	95.00 (80.00–100.00)	0.00 (0.00–20.00)	—	—
Minimum–maximum (%)	25.00–100.00	15.00–100.00	−30.00–55.00	—	—
Wilcoxon signed-rank ranks	9 negative, 21 positive, 14 ties	—	—	W = 106.50	—
Effect size, r	—	—	—	0.39	—

Scores are presented as percentages. Change represents post-test minus baseline score in percentage points. The pre-post comparison was performed using the Wilcoxon signed-rank test. Effect size was calculated as |Z|/√N using N = 44.

**FIGURE 2:**
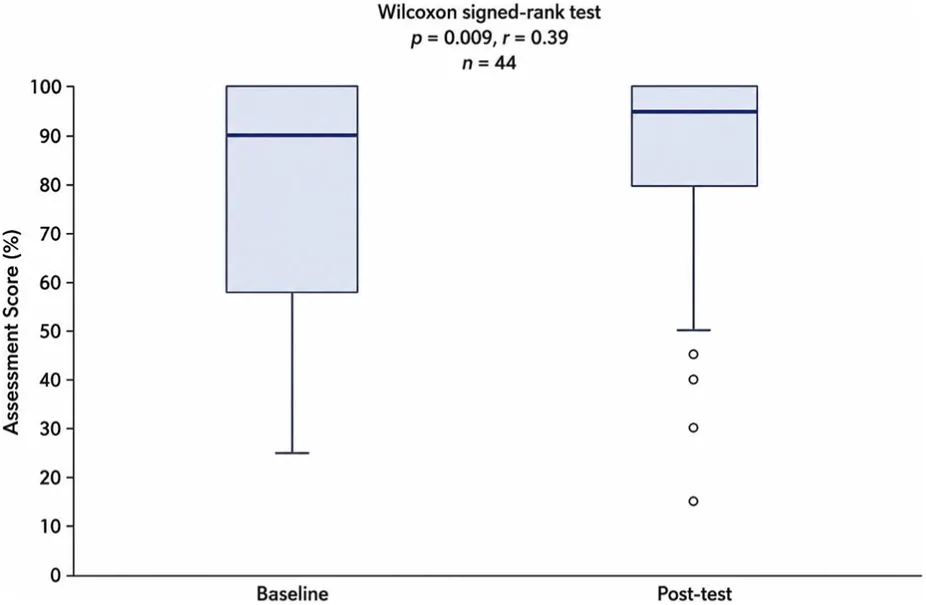
Pre- and post-intervention assessment scores. Boxplots showing baseline and post-test total assessment scores among participating students (n = 44). Scores are presented as percentages. Post-test scores were significantly higher than baseline scores using the Wilcoxon signed-rank test (Z = −2.597, p = 0.009, r = 0.39). Open circles indicate outlying observations identified using the standard boxplot criterion of values more than 1.5 interquartile ranges beyond the box boundary.

### Domain-specific learning outcomes

Domain-specific analyses showed significant improvements in platelet pharmacology and coagulation pharmacology, while the increase in experimental reasoning did not reach statistical significance. Platelet pharmacology scores increased from 3.95 ± 1.22 to 4.39 ± 1.10 out of 5, with a median increase from 4.00 to 5.00. This improvement was statistically significant (Z = −1.975, p = 0.048, r = 0.30). Coagulation pharmacology showed the largest domain-specific improvement, increasing from 3.82 ± 1.35 to 4.55 ± 0.85 out of 5, with a statistically significant Wilcoxon result (Z = −3.281, p = 0.001, r = 0.49). Experimental reasoning scores increased from 7.75 ± 2.83 to 8.23 ± 2.84 out of 10; however, this change was not statistically significant (Z = −1.228, p = 0.219, r = 0.19). Domain-specific outcomes are summarized in Table 3 and shown graphically in Figure 3.

**TABLE 3:** Domain-specific pre- and post-intervention assessment scores.

Domain	Baseline mean ± SD	Post-test mean ± SD	Mean change	Baseline median (IQR)	Post-test median (IQR)	Negative/positive/tied ranks	Z	p-value	Effect size r
Platelet pharmacology score/5	3.95 ± 1.22	4.39 ± 1.10	+0.44	4.00 (3.00–5.00)	5.00 (4.00–5.00)	7/17/20	−1.975	0.048	0.30
Coagulation pharmacology score/5	3.82 ± 1.35	4.55 ± 0.85	+0.73	4.00 (2.25–5.00)	5.00 (4.00–5.00)	4/19/21	−3.281	0.001	0.49
Experimental reasoning score/10	7.75 ± 2.83	8.23 ± 2.84	+0.48	9.00 (6.00–10.00)	10.00 (8.00–10.00)	9/17/18	−1.228	0.219	0.19

Scores are presented using their original domain scales. Change represents post-test minus baseline score. Pre-post comparisons were performed using Wilcoxon signed-rank tests. Effect size was calculated as |Z|/√N using N = 44.

**FIGURE 3:**
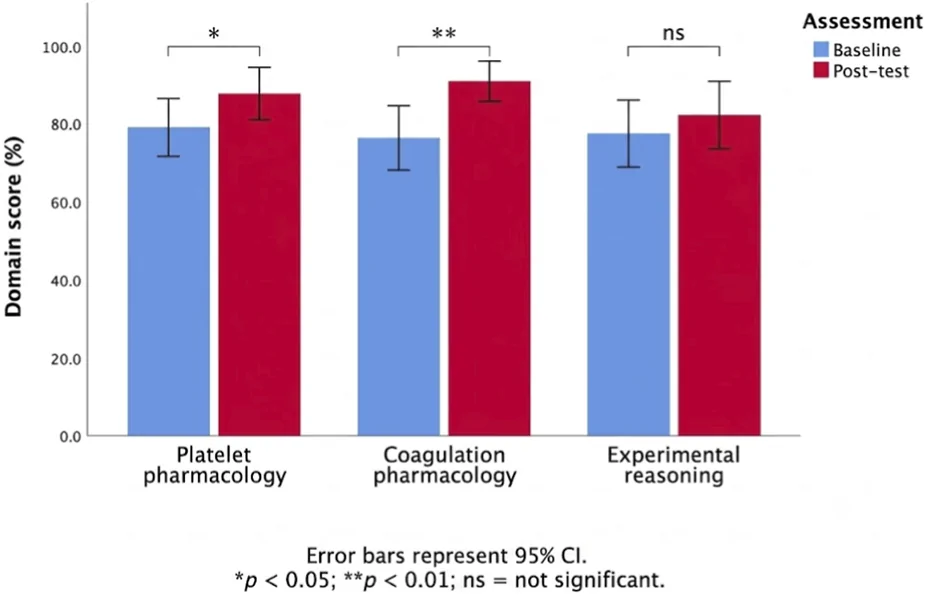
Domain-specific pre- and post-intervention assessment scores. Bar charts showing mean baseline and post-test scores for platelet pharmacology, coagulation pharmacology, and experimental reasoning domains. Scores are presented as percentages to allow comparison across domains with different maximum scores. Error bars represent 95% confidence intervals. Pre-post comparisons were performed using Wilcoxon signed-rank tests. Platelet pharmacology and coagulation pharmacology improved significantly, whereas experimental reasoning did not reach statistical significance. Analyses include all students with paired baseline and post-test data (n = 44).

### Performance during the AI-supported mechanistic reasoning practical

Forty-three students completed the Step 3 AI-supported mechanistic reasoning practical. The mean total reasoning score was 17.88 ± 4.01 out of 21, equivalent to 85.1% ± 19.1%. The median score was 20.00 out of 21 (IQR 15.00–21.00), indicating generally high performance during the practical.

When performance was examined by reasoning component, students achieved the highest accuracy in experimental interpretation, with a mean score of 6.09 ± 1.13 out of 7, equivalent to 87.0% ± 16.1%. Mechanism/pathway identification scores averaged 5.95 ± 1.54 out of 7, equivalent to 85.0% ± 22.0%. Drug-class identification scores averaged 5.84 ± 1.65 out of 7, equivalent to 83.4% ± 23.5%. Median scores for mechanism/pathway identification and drug-class identification were both 7.00 out of 7, suggesting that many students achieved full scores in these components.

Students also reported high confidence during the practical, with a mean confidence score of 4.00 ± 0.96 out of 5 and a median of 4.14 (IQR 3.14–5.00). The mean percentage of cases in which students reported that AI helped their reasoning was 94.0% ± 19.8%, with a median of 100.0%. Step 3 practical performance is summarized in Table 4, and component-level accuracy is shown in Figure 4.

**TABLE 4:** Performance during the Step 3 AI-supported mechanistic reasoning practical.

Measure	Mean ± SD	Median (IQR)	Minimum–maximum
Step 3 total reasoning score/21	17.88 ± 4.01	20.00 (15.00–21.00)	7–21
Step 3 total reasoning score, %	85.1 ± 19.1	95.2 (71.4–100.0)	33.3–100.0
Experimental interpretation score/7	6.09 ± 1.13	6.00 (5.00–7.00)	2–7
Experimental interpretation score, %	87.0 ± 16.1	85.7 (71.4–100.0)	28.6–100.0
Mechanism/pathway identification score/7	5.95 ± 1.54	7.00 (5.00–7.00)	1–7
Mechanism/pathway identification score, %	85.0 ± 22.0	100.0 (71.4–100.0)	14.3–100.0
Drug-class identification score/7	5.84 ± 1.65	7.00 (5.00–7.00)	1–7
Drug-class identification score, %	83.4 ± 23.5	100.0 (71.4–100.0)	14.3–100.0
Mean confidence during step 3 practical/5	4.00 ± 0.96	4.14 (3.14–5.00)	1.57–5.00
AI helpfulness, % of cases	94.0 ± 19.8	100.0 (100.0–100.0)	0.0–100.0

Analyses include students who completed the Step 3 AI-supported practical (n = 43). One student had missing Step 3 data. The practical consisted of seven simulated cases, with each component score calculated across the seven cases. AI, helpfulness represents the percentage of Step 3 cases in which the student reported that AI, helped their reasoning. The Step 3 practical used structured questions on experimental interpretation, pathway/mechanism identification, drug-class identification, confidence, and perceived AI, usefulness.

**FIGURE 4:**
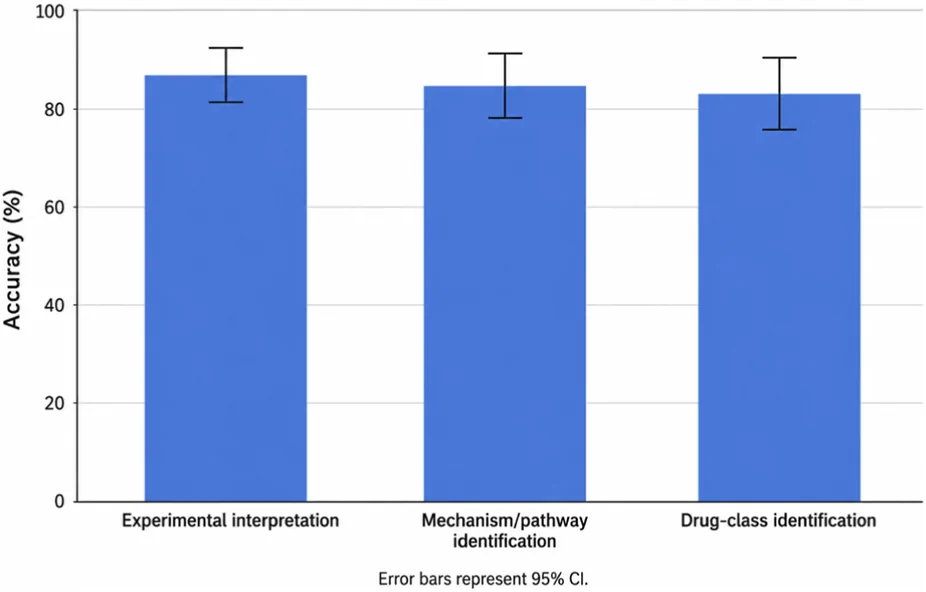
Accuracy of core reasoning components during the AI-supported practical. Bar chart showing student performance in experimental interpretation, mechanism/pathway identification, and drug-class identification during the Step 3 AI-supported mechanistic reasoning practical. Scores are presented as percentages of the maximum possible score for each component. Error bars represent 95% confidence intervals. Analyses include students who completed Step 3 (n = 43).

### Student perceptions and acceptability

The 10 core perception and acceptability items demonstrated excellent internal consistency reliability, with Cronbach’s alpha of 0.935. The two safety/limitations items concerning AI-related confusion and the need for instructor guidance were analyzed separately. Overall, students reported favorable perceptions of the AI-guided mechanistic reasoning practical. Mean item scores ranged from 3.70 ± 0.93 to 4.18 ± 0.66 on the five-point Likert scale.

Agreement was highest for improved understanding of anticoagulant mechanisms and for the usefulness of AI without simply providing answers, with 38 students (86.4%) agreeing or strongly agreeing with each of these items. High levels of agreement were also observed for interpreting platelet aggregation curves (37/44, 84.1%), improved data-to-mechanism reasoning (37/44, 84.1%), and support for future use of AI-guided practicals (37/44, 84.1%). Agreement with items related to antiplatelet mechanisms and connecting mechanisms to experimental readouts was 79.5% for both items.

The lowest agreement was observed for the AI-related interpretation/confusion item (32/44, 72.7%) and the item indicating that instructor guidance remained necessary (34/44, 77.3%). These perception results are summarized in Table 5 and visualized in Figure 5.

**TABLE 5:** Student perceptions of the AI-guided mechanistic reasoning practical.

Survey item	Mean ± SD	Median (IQR)	Agree/strongly agree, n (%)
Improved understanding of antiplatelet mechanisms	4.07 ± 0.76	4.00 (4.00–5.00)	35 (79.5)
Improved understanding of anticoagulant mechanisms	4.18 ± 0.66	4.00 (4.00–5.00)	38 (86.4)
Helped connect mechanisms to experimental readouts	4.07 ± 0.76	4.00 (4.00–5.00)	35 (79.5)
Helped interpret platelet aggregation curves	4.05 ± 0.78	4.00 (4.00–4.75)	37 (84.1)
Helped interpret PT/aPTT coagulation findings	4.07 ± 0.73	4.00 (4.00–5.00)	36 (81.8)
Improved data-to-mechanism reasoning	4.14 ± 0.67	4.00 (4.00–5.00)	37 (84.1)
Helped identify pathway before drug class	4.09 ± 0.74	4.00 (4.00–5.00)	36 (81.8)
Increased confidence interpreting experimental data	4.09 ± 0.88	4.00 (4.00–5.00)	36 (81.8)
AI was useful without simply giving answers	4.14 ± 0.63	4.00 (4.00–5.00)	38 (86.4)
Support future use of AI-guided practicals	4.14 ± 0.67	4.00 (4.00–5.00)	37 (84.1)
AI-related interpretation/confusion item	3.70 ± 0.93	4.00 (3.00–4.00)	32 (72.7)
Instructor guidance remains necessary	3.84 ± 0.89	4.00 (4.00–4.00)	34 (77.3)

**FIGURE 5:**
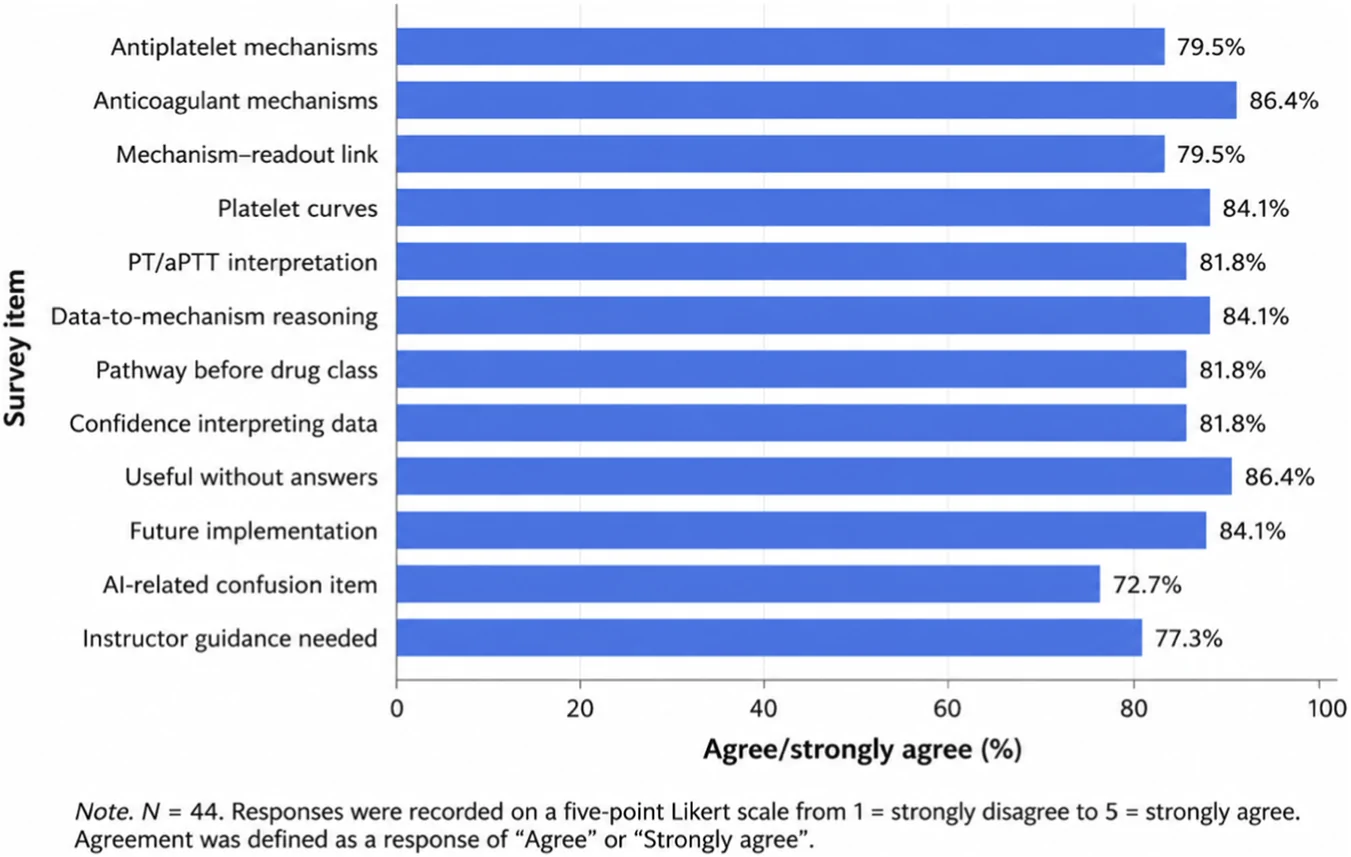
Student perceptions of the AI-guided mechanistic reasoning practical. Horizontal bar chart showing the percentage of students who agreed or strongly agreed with each perception survey item. Responses were recorded on a five-point Likert scale. Agreement was defined as a response of “Agree” or “Strongly agree.”

### Associations between practical performance and learning outcomes

Spearman correlation analyses showed that Step 3 practical performance was positively associated with post-test achievement. The Step 3 total reasoning score was positively correlated with post-test total score (ρ = 0.606, p < 0.001, n = 43). Step 3 performance was also weakly to moderately associated with baseline total score (ρ = 0.330, p = 0.031, n = 43), but it was not significantly associated with learning gain (ρ = 0.206, p = 0.186, n = 43).

Baseline total score was negatively associated with learning gain (ρ = −0.583, p < 0.001, n = 44). Mean confidence during Step 3 was positively correlated with Step 3 total reasoning score (ρ = 0.545, p < 0.001, n = 43). AI helpfulness was also weakly to moderately associated with Step 3 performance (ρ = 0.317, p = 0.038, n = 43). These associations are presented in Table 6.

**TABLE 6:** Associations between AI-supported practical performance and learning outcomes.

Comparison	Spearman’s ρ	p-value	N	Interpretation
Step 3 total reasoning score (%) vs. post-test total score (%)	0.606	<0.001	43	Moderate-to-strong positive association
Step 3 total reasoning score (%) vs. baseline total score (%)	0.330	0.031	43	Weak-to-moderate positive association
Step 3 total reasoning score (%) vs. learning gain (%)	0.206	0.186	43	Not statistically significant
Baseline total score (%) vs. learning gain (%)	−0.583	<0.001	44	Moderate negative association
Mean confidence during step 3 vs. step 3 total reasoning score (%)	0.545	<0.001	43	Moderate positive association
AI helpfulness (%) vs. step 3 total reasoning score (%)	0.317	0.038	43	Weak-to-moderate positive association

Correlations were assessed using Spearman’s rank correlation coefficient. Step 3-related analyses included students who completed the AI-supported practical (n = 43). The baseline score *versus* learning gain analysis included all students with baseline and post-test data (n = 44). Learning gain was calculated as post-test total score minus baseline total score.

## Discussion

This study found that a structured, multi-component experimental pharmacology practical that included an AI-guided reasoning prompt was associated with improved short-term assessment scores and favorable student perceptions. The intervention combined an experimental logic mini-brief, repeated case-based reasoning, active answer generation, and AI-supported prompting. Therefore, the findings should be interpreted as outcomes following an integrated instructional activity rather than evidence that AI alone produced the observed gains. The intended value of the AI component was to place student interaction within a guided reasoning sequence moving from experimental finding to affected pathway, mechanism, and drug class.

The improvement in overall assessment scores supports further evaluation of AI-guided learning when it is tied to a specific educational task. Mean total scores increased from 76.82% at baseline to 85.80% after the intervention, with a moderate effect size. This finding is important because much of the early literature on generative AI in health professions education has focused on possible uses, student views, and concerns about assessment integrity rather than measured learning outcomes (Preiksaitis and Rose, 2023; Patino et al., 2024). The present study adds short-term outcome data from a practical pharmacology setting. It also supports recent work suggesting that AI may help learning when it is built into a structured activity rather than used as an open source of answers (Sridharan and Sequeira, 2024; Feigerlova et al., 2025).

The magnitude and distribution of the change are also important. The mean improvement was 8.98 percentage points with a moderate effect size (r = 0.39), suggesting a moderate short-term group-level shift. Improvement was not uniform, however: 21 students improved, 14 had unchanged scores, and 9 scored lower on the post-test. The findings therefore indicate a group-level improvement following the intervention rather than a universal benefit for all learners.

The strongest domain-level improvement was seen in coagulation pharmacology. One possible explanation is that PT and aPTT interpretation follows a clearer pathway logic than some platelet aggregation tasks. Students can link a prolonged PT to the extrinsic pathway, a prolonged aPTT to the intrinsic pathway, and changes in both tests to shared pathway effects. Future studies could test whether coagulation cases are especially suitable for stepwise AI-guided reasoning. Platelet pharmacology also improved, but the gain was smaller. This may reflect the high baseline score in this domain and the greater complexity of platelet aggregation, where activation, amplification, thromboxane signaling, ADP signaling, and GP IIb/IIIa function interact. Learning to interpret platelet aggregation curves may require more repeated practice than a single session can provide.

Experimental reasoning scores increased numerically but did not reach statistical significance. This null result is important because experimental reasoning was a central target of the intervention. The baseline median was already 9 out of 10 and increased to 10 out of 10 after the intervention, leaving limited room for measurable improvement. The small effect size (r = 0.19) also suggests that any short-term change in this broader reasoning construct was modest. Several explanations remain possible, including ceiling effects, limited sensitivity of the 10-item reasoning measure, insufficient power to detect a small change, or a true lack of measurable improvement after a single session. The pattern may also indicate that one practical session can reinforce domain-specific knowledge more readily than a broader reasoning skill, which may require repeated practice across different experimental contexts.

Performance during the AI-supported practical was high. Students achieved a mean Step 3 reasoning score of 85.1%, with the highest accuracy in experimental interpretation, followed by mechanism or pathway identification and drug-class identification. This pattern is consistent with the intended reasoning sequence, in which students first interpret the experimental finding and then move toward pathway and drug-class inference. Because Step 3 was completed with AI support, these scores should be interpreted as performance during an AI-supported task rather than as independent reasoning performance alone. Drug-class identification was slightly lower, which is expected because it requires an extra step from mechanism to pharmacological category.

The observed score improvement should be interpreted in the context of the multi-component design. The intervention included a mini-brief, repeated case-based practice, active answer generation, and an AI-guided reasoning prompt. Each component could have contributed to the post-test gains. In particular, the 21 scored questions completed during the practical may have acted as retrieval practice, and the mini-brief re-exposed students to content that was later assessed. The present design therefore cannot determine the independent contribution of AI guidance.

The correlation results should be interpreted cautiously. Step 3 performance was associated with post-test score, but it was also associated with baseline score and was not significantly associated with learning gain. This pattern suggests that the Step 3-post-test association may partly reflect prior pharmacology knowledge rather than a distinct learning effect of the practical. Because Step 3 was completed with AI support, these scores also reflect performance in an AI-supported task rather than independent student reasoning alone. The negative association between baseline score and learning gain should also be interpreted cautiously because gain scores are mathematically coupled to baseline values and may be influenced by regression to the mean.

The student perception data support the acceptability of this approach. Most students agreed that the activity improved their understanding of antiplatelet and anticoagulant mechanisms, helped them connect mechanisms to experimental readouts, and supported future use of AI-guided practicals. The high agreement that AI was useful without simply giving answers is especially important. It suggests that students perceived the AI as a reasoning aid rather than only as an answer source. Positive and negative perceptions were not mutually exclusive. Although 86.4% of students agreed that AI was useful without simply providing answers, 72.7% also agreed that AI sometimes made the reasoning process more confusing. The relatively small difference between these proportions suggests that students could perceive AI as useful while still experiencing uncertainty during the reasoning process. Acceptability should therefore not be interpreted as uniformly positive. This distinction matters because unguided AI use may promote passive learning, overreliance, and reduced student agency (Darvishi et al., 2024; Fan et al., 2024).

Instructor guidance remains central. Student agreement with the need for instructor guidance should not be interpreted as a weakness of the intervention. It supports the need for teacher oversight. AI can help students ask better questions and test their reasoning, but it should not replace the teacher’s role in setting the task, defining standards, correcting errors, and linking the activity to course outcomes. In experimental pharmacology, where students must connect laboratory data to physiology and drug action, teacher oversight is still needed.

The study cannot confirm how AI was used in every interaction. Students were instructed to use AI as a reasoning aid and not to request direct answers, but interaction logs, transcripts, or screen recordings were not collected. The study therefore cannot verify that AI consistently acted as a reasoning scaffold or that students did not obtain direct answers during the practical. Future controlled studies should archive AI interactions so that intervention fidelity, prompting behavior, and possible answer leakage can be examined directly.

The characteristics of the cohort should also be considered when interpreting the findings. Most participants were aged 36 years or older, 72.7% reported previous hospital pharmacy work experience, 72.7% had previously used AI for pharmacology learning, and 61.4% had used AI to interpret data, graphs, or laboratory results. The cohort also had relatively high self-reported GPA values. These characteristics may have increased familiarity with both pharmacology and AI-supported learning and may limit generalizability to younger, less experienced, or less AI-familiar entry-level PharmD cohorts.

This study has several limitations. First, the single-group pre-post design does not allow causal inference. Without a control group, score gains cannot be attributed specifically to the AI-guided component. The mini-brief, case-based practice, content re-exposure, retrieval practice, and test familiarity may all have contributed to the observed improvement (Anastasi and Capili, 2024). A further interpretive issue is that the pretest may itself have contributed to later performance gains through retrieval practice or item/content re-exposure, independent of the AI interaction (Roediger and Karpicke, 2006; Roediger and Butler, 2011). The next step should directly compare the structured AI-guided practical with an otherwise equivalent non-AI condition, with the instructional content, assessment exposure, and practice time held constant. Second, AI interaction logs were not collected, so fidelity to the intended guided-reasoning format could not be verified. Variation in the AI systems used by students represents an additional source of heterogeneity. Differences in model version, subscription tier, personalization settings, and prior user familiarity may have affected the quality and style of AI responses despite use of a common instructional prompt. Generative AI systems are also updated frequently, meaning that the behavior of models available during the study period may differ from later versions and limit the temporal reproducibility of AI-supported educational interventions. Third, the post-test was administered immediately after the intervention, so the study did not assess long-term retention. Fourth, the cohort also had relatively high baseline performance, with most students reporting GPA values in the upper part of the scale. Together with the high baseline assessment scores, this may have contributed to ceiling effects and may limit generalizability to cohorts with different levels of prior academic performance. Fifth, domain-specific analyses were exploratory and were not adjusted for multiple comparisons; therefore, the platelet pharmacology result should be interpreted cautiously. Finally, the cohort included mature students with substantial prior pharmacy experience, which may limit generalizability to entry-level PharmD students.

A structured experimental pharmacology practical that combined a mini-brief, repeated case-based reasoning, and an AI-guided prompt was feasible, acceptable to students, and associated with improved short-term assessment scores. The findings support further study of AI-guided reasoning activities in pharmacology education, but they do not establish that AI independently caused the observed learning gains. Future controlled studies should isolate the AI component, include delayed post-tests, and examine AI interaction logs to assess how students use AI during mechanistic reasoning tasks.

## Data Availability

The raw data supporting the conclusions of this article will be made available by the authors, without undue reservation.
