# Supplementary material for: Evaluation of a multi-component AI-guided mechanistic reasoning practical in experimental pharmacology: a single-group pre-post study
Source: Front Pharmacol. 2026 Sep 21;17:1937176. doi: 10.3389/fphar.2026.1937176 (PMC13634817; doi:10.3389/fphar.2026.1937176)
Supplement: Supplementary file 1 [file DataSheet1.pdf]

Step 1: Baseline Assessment

The baseline assessment should measure students' **starting knowledge and reasoning before the AI-supported practical** and allow paired comparison with the post-intervention assessment. Estimated completion time: 15–20 minutes.

* Required

Demographics

1. Please enter your anonymous study ID. *

Select your answer

2. Specify your age *

Select your answer

3. Self-reported GPA range *

Select your answer

A. Baseline Knowledge Test. 20 marks total

Domain 1: Platelet Pharmacology knowledge
This assessment is intended to evaluate your current baseline knowledge before the practical session. Please complete the assessment independently without using AI tools, online searches, notes, or external assistance.

4. Q1. Which receptor is mainly responsible for ADP-mediated amplification of platelet aggregation? * (1 Point)

- A. P2Y12 receptor
- B. β1 receptor
- C. H1 receptor
- D. D2 receptor

5. Q2. Aspirin inhibits platelet aggregation mainly by blocking: * (1 Point)

- A. ADP binding to P2Y12 receptors
- B. Cyclooxygenase-1–mediated thromboxane A_2 synthesis
- C. Fibrinogen binding to GP IIb/IIIa
- D. Thrombin binding to antithrombin

6. Q3. GP IIb/IIIa inhibitors reduce platelet aggregation by preventing: * (1 Point)

- A. Platelet adhesion to collagen
- B. ADP release from dense granules
- C. Fibrinogen-mediated platelet cross-linking
- D. Conversion of fibrinogen to fibrin

7. Q4. In light transmission aggregometry, increased platelet aggregation usually causes: * (1 Point)

- A. Decreased light transmission
- B. Increased light transmission
- C. No change in light transmission
- D. Complete sample clotting

8. Q5. A platelet aggregation curve showing an initial response followed by loss of sustained aggregation is most consistent with impaired: * (1 Point)

- ◯ A. Primary platelet adhesion only
- ◯ B. ADP-mediated amplification
- ◯ C. Fibrin formation
- ◯ D. Vitamin K recycling

Domain 2: Coagulation pharmacology knowledge

9. Q6. Prothrombin time mainly assesses the: * (1 Point)

- A. Intrinsic and common pathways
- B. Extrinsic and common pathways
- C. Platelet adhesion pathway
- D. Fibrinolytic pathway

10. Q7. Activated partial thromboplastin time mainly assesses the: * (1 Point)

- A. Extrinsic pathway only
- B. Intrinsic and common pathways
- C. Platelet aggregation pathway
- D. Thromboxane pathway

11. Q8. Warfarin prolongs coagulation mainly by reducing the activity of: * (1 Point)

- A. Factors II, VII, IX, and X
- B. Factors I, V, VIII, and XIII
- C. Platelet P2Y12 receptors
- D. Antithrombin III

12. Q9. Unfractionated heparin produces its anticoagulant effect mainly by enhancing the activity of: * (1 Point)

- A. Vitamin K epoxide reductase
- B. Antithrombin
- C. Cyclooxygenase-1
- D. GP IIb/IIIa

13. Q10. A drug that directly inhibits factor Xa would be expected to interfere mainly with: * (1 Point)

- A. Platelet adhesion only
- B. The common coagulation pathway
- C. Thromboxane synthesis only
- D. Fibrinolysis only

Domain 3: Experimental reasoning

14. Q11. A sample has markedly prolonged aPTT with relatively normal PT. This pattern most strongly suggests interference with the: * (1 Point)

- A. Extrinsic pathway
- B. Intrinsic pathway
- C. Platelet aggregation pathway
- D. Thromboxane pathway

15. Q12. A drug abolishes aggregation regardless of whether ADP, collagen, or thrombin is used as the agonist. The most likely target is: * (1 Point)

- A. COX-1
- B. P2Y12 receptor
- C. GP IIb/IIIa receptor
- D. Vitamin K epoxide reductase

16. **Q13.** If arachidonic acid fails to induce platelet aggregation, but thrombin still induces aggregation, the most likely inhibited target is: * (1 Point)

- A. COX-1/thromboxane pathway
- B. GP IIb/IIIa receptor
- C. Factor Xa
- D. Antithrombin

17. **Q14.** A student concludes that a drug is warfarin because PT is prolonged more than aPTT. This conclusion is based mainly on the sensitivity of PT to:
* (1 Point)

- A. Factor VII depletion
- B. Platelet ADP release
- C. Fibrinogen binding to GP IIb/IIIa
- D. Thrombin receptor activation

18. **Q15.** In an experimental pharmacology practical, the strongest evidence for identifying a drug mechanism comes from: * (1 Point)

- A. The drug name
- B. The color of the sample
- C. The pattern of change in the curve
- D. The student's previous lecture notes

5 case-based interpretation questions × 1 mark = 5 marks

19. **Case 1: Platelet curve interpretation.** A platelet sample is stimulated with ADP. The curve shows a small initial rise followed by failure to maintain aggregation. The most likely mechanism is: * (1 Point)

- ◯ COX-1 inhibition
- ◯ P2Y12 receptor inhibition
- ◯ Factor Xa inhibition
- ◯ Vitamin K antagonism

20. **Case 2: Final common pathway inhibition.** A platelet sample shows almost no aggregation after stimulation with ADP, collagen, and thrombin.The most likely drug class is: * (1 Point)

- ◯ A. Aspirin-like COX inhibitor
- ◯ B. P2Y12 receptor antagonist
- ◯ C. GP IIb/IIIa inhibitor
- ◯ D. Direct thrombin inhibitor

21. **Case 3: Agonist sensitivity.** A platelet sample fails to aggregate in response to arachidonic acid but still aggregates in response to thrombin.The most likely inhibited pathway is: * (1 Point)

- ◯ A. COX-1/thromboxane A_2 pathway
- ◯ B. P2Y12 receptor pathway
- ◯ C. GP IIb/IIIa receptor pathway
- ◯ D. Intrinsic coagulation pathway

22. **Case 4: Coagulation test interpretation.** A plasma sample shows: PT Markedly prolonged, aPTT Mildly prolonged. The most likely drug effect is: * (1 Point)

- ◯ A. Warfarin-like vitamin K antagonism
- ◯ B. Aspirin-like COX inhibition
- ◯ C. P2Y12 receptor antagonism
- ◯ D. GP IIb/IIIa inhibition

23. **Case 5: aPTT-dominant prolongation.** A plasma sample shows:PTNormal or mildly prolongedaPTTMarkedly prolongedThe most likely mechanism is: * (1 Point)

- A. Selective platelet COX-1 inhibition
- B. Antithrombin-mediated inhibition of thrombin/factor Xa
- C. Blockade of platelet ADP receptors
- D. Inhibition of fibrinogen binding to GP IIb/IIIa

24. How confident are you in your answers? *

- Extremely confident
- Somewhat confident
- Neutral
- Somewhat not confident
- Extremely not confident

Educational and AI Exposure Variables

25. Have you previously studied anti-platelet drugs in detail? *

- Yes
- No
- Not sure

26. Have you previously studied anticoagulant drugs in detail? *

- Yes
- No
- Not sure

27. Did you attend the relevant cardiovascular pharmacology lectures before this practical? *

- Yes
- No
- Partially

28. Do you have previous pharmacy work experience? *

- No
- Community Pharmacy
- Hospital Pharmacy
- Other healthcare setting

29. How confident are you in interpreting pharmacology laboratory data? *

- 1 = Not confident
- 2 = Slightly confident
- 3 = Moderately confident
- 4 = Confident
- 5 = Very confident

AI familiarity/confidence

30. How often do you use AI tools such as ChatGPT, Gemini, or Copilot for studying? *

- ◯ Never
- ◯ Rarely
- ◯ Sometimes
- ◯ Often
- ◯ Very often

31. Have you previously used AI to understand pharmacology mechanisms? *

- ◯ Yes
- ◯ No

32. Have you previously used AI to interpret data, graphs, or lab results? *

- ◯ Yes
- ◯ No

33. How confident are you in using AI for academic learning? *

- ◯ 1 = Not confident
- ◯ 2 = Slightly confident
- ◯ 3 = Moderately confident
- ◯ 4 = Confident
- ◯ 5 = Very confident

End of Baseline assessment

This content is neither created nor endorsed by Microsoft. The data you submit will be sent to the form owner.

Microsoft Forms
