# Supplementary material for: Evaluation of a multi-component AI-guided mechanistic reasoning practical in experimental pharmacology: a single-group pre-post study
Source: Front Pharmacol. 2026 Sep 21;17:1937176. doi: 10.3389/fphar.2026.1937176 (PMC13634817; doi:10.3389/fphar.2026.1937176)
Supplement: Supplementary file 2 [file DataSheet2.pdf]

“Experimental Logic in Antithrombotic Pharmacology”

Platelet Function & Antiplatelet Drugs

Simulation Lab: From Molecular Targets to Aggregation Curves

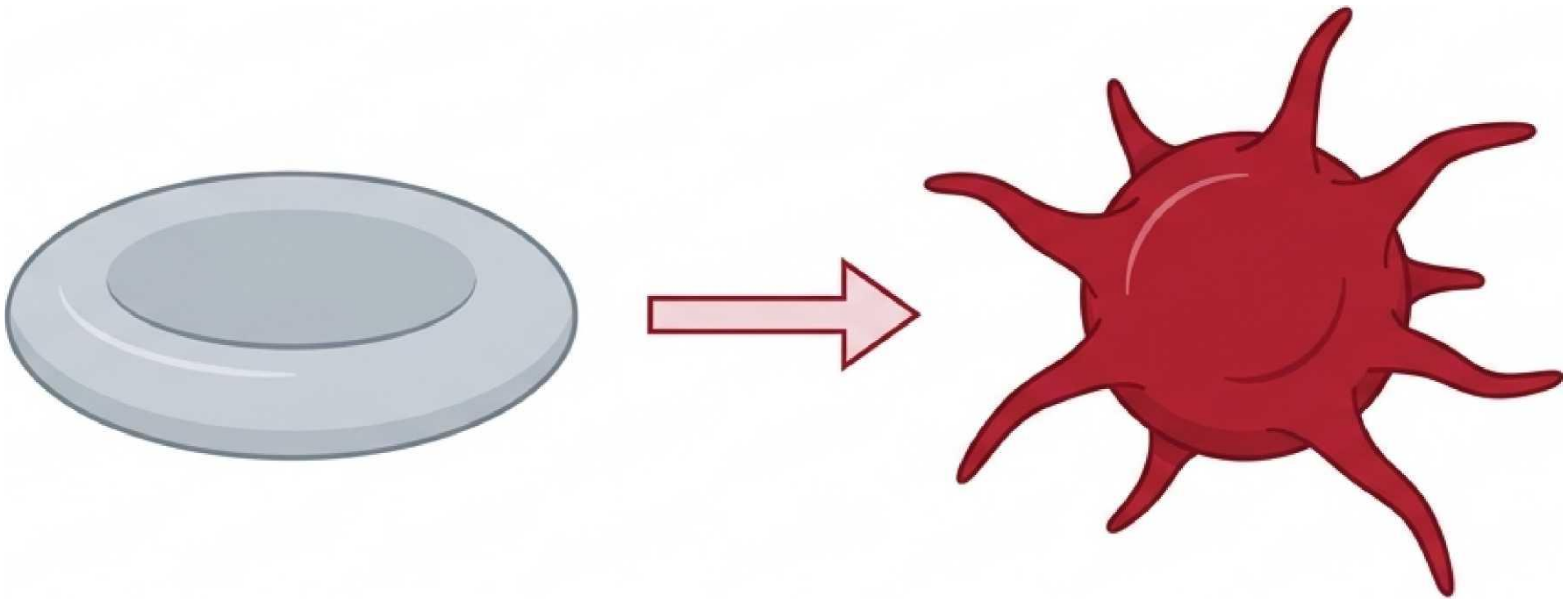

Second Year PharmD Students

From Receptor Theory to Experimental Data

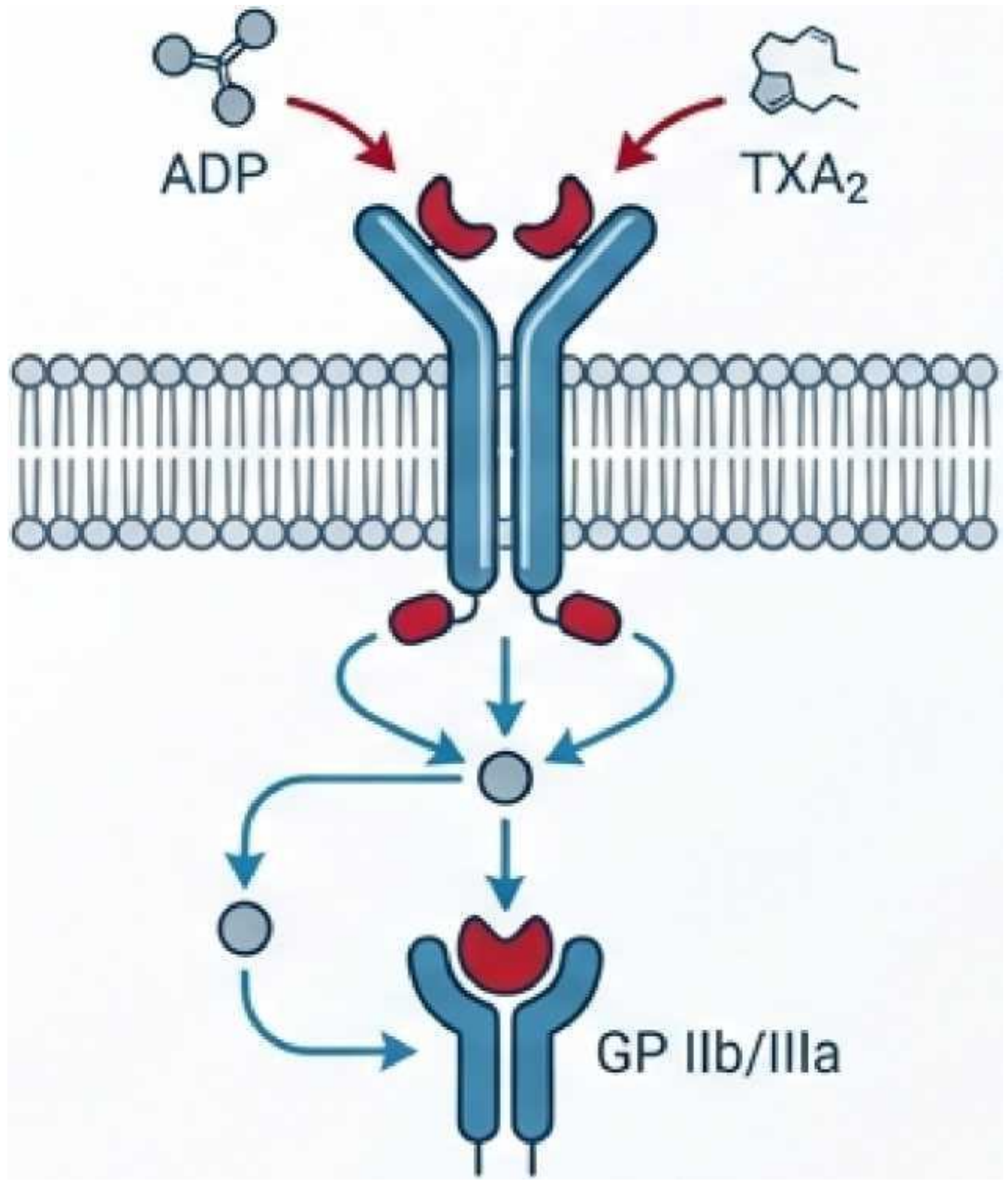

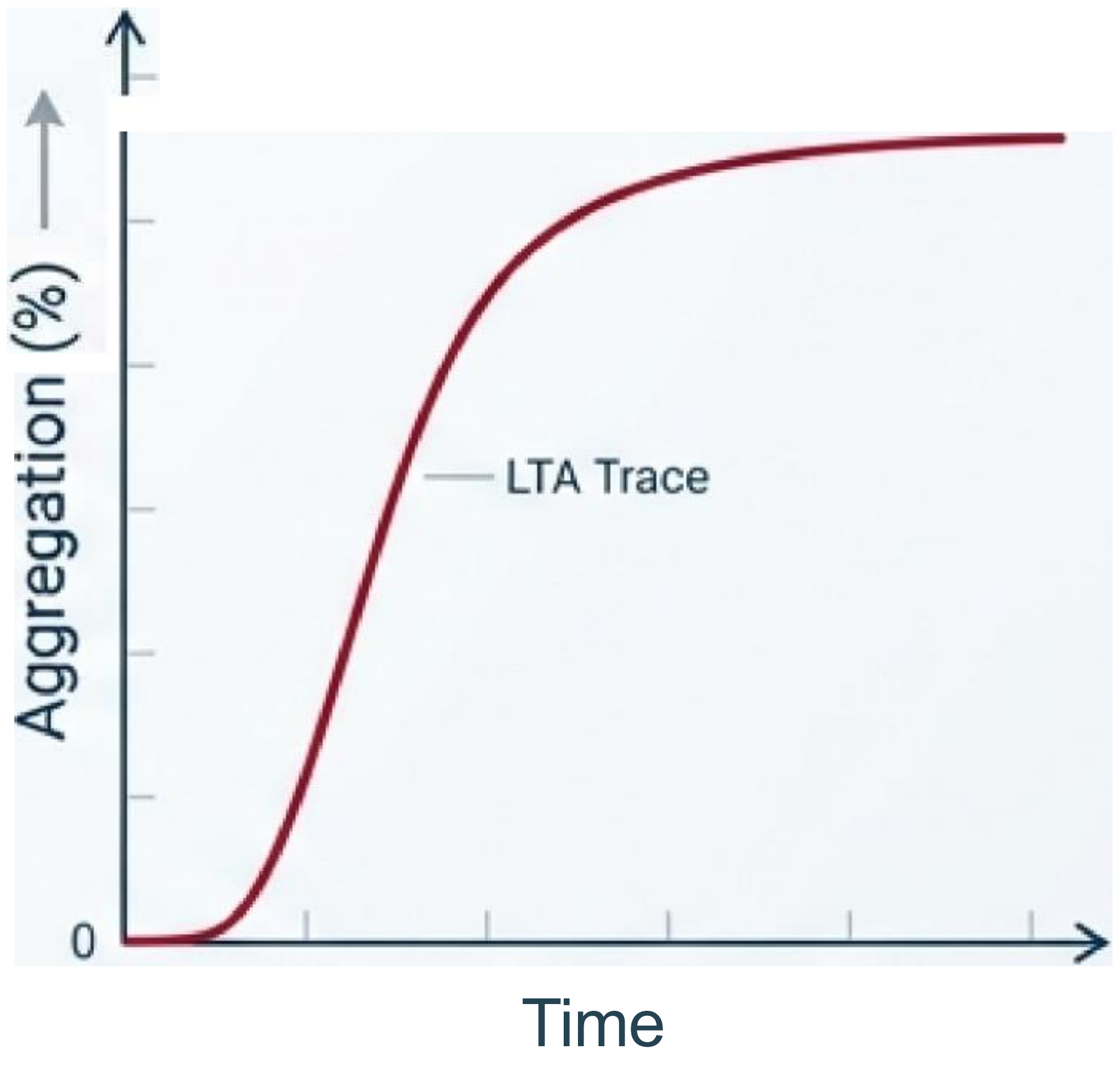

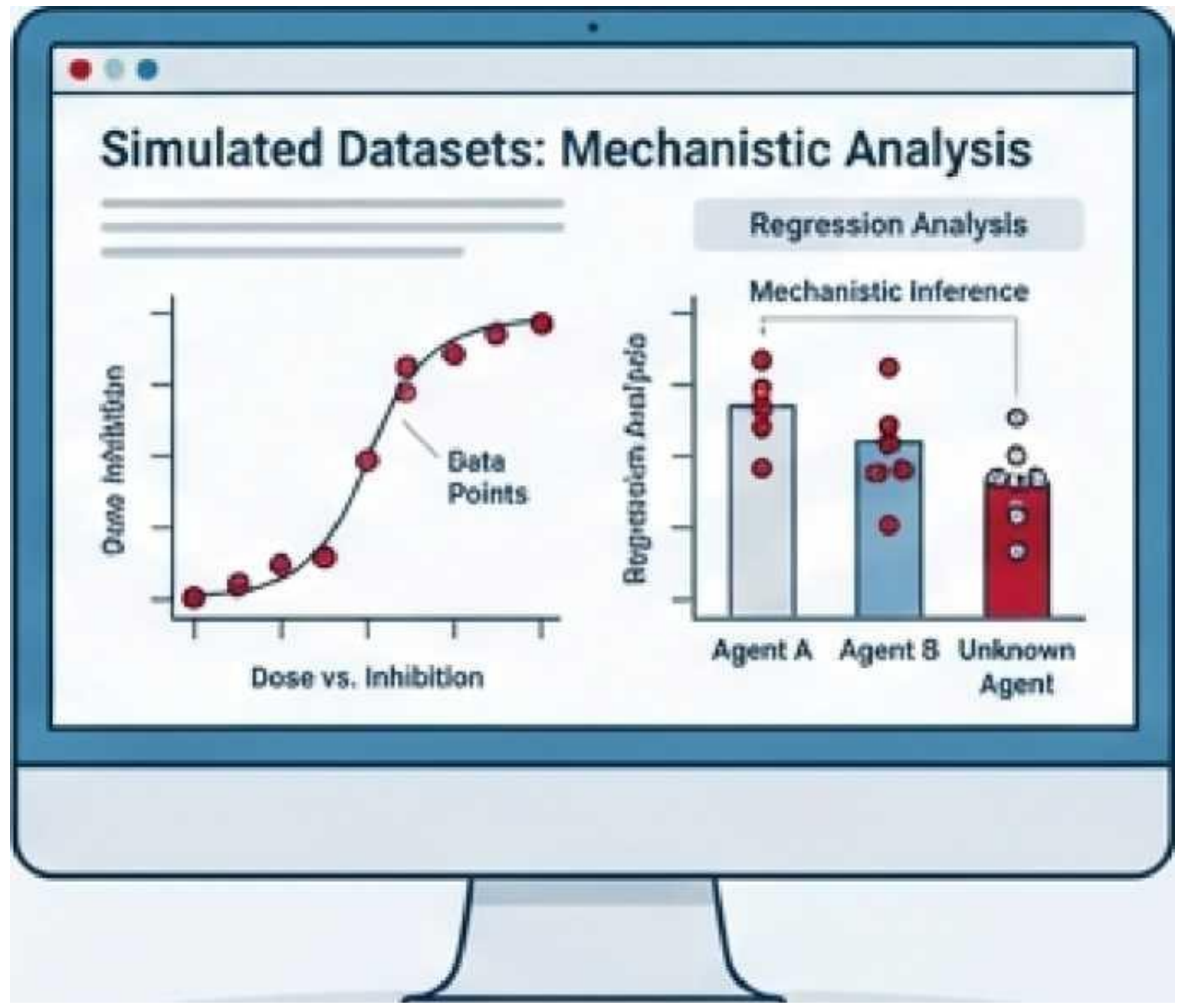

1. Map Pathways, Connect physiological inputs (ADP/TXA2) to the Final Common Pathway (GP IIb/IIIa).

2. Interpret Curves. Read Light Transmission Aggregometry (LTA) plots as a direct measure of pharmacological activity.

3. Analyze Simulation. Use simulated datasets to deduce the mechanistic target of unknown agents.

Focus: Today is about mechanistic inference. We deduce **how** a drug works by observing **how it changes the curve.**

The Physiology of Activation and Amplification

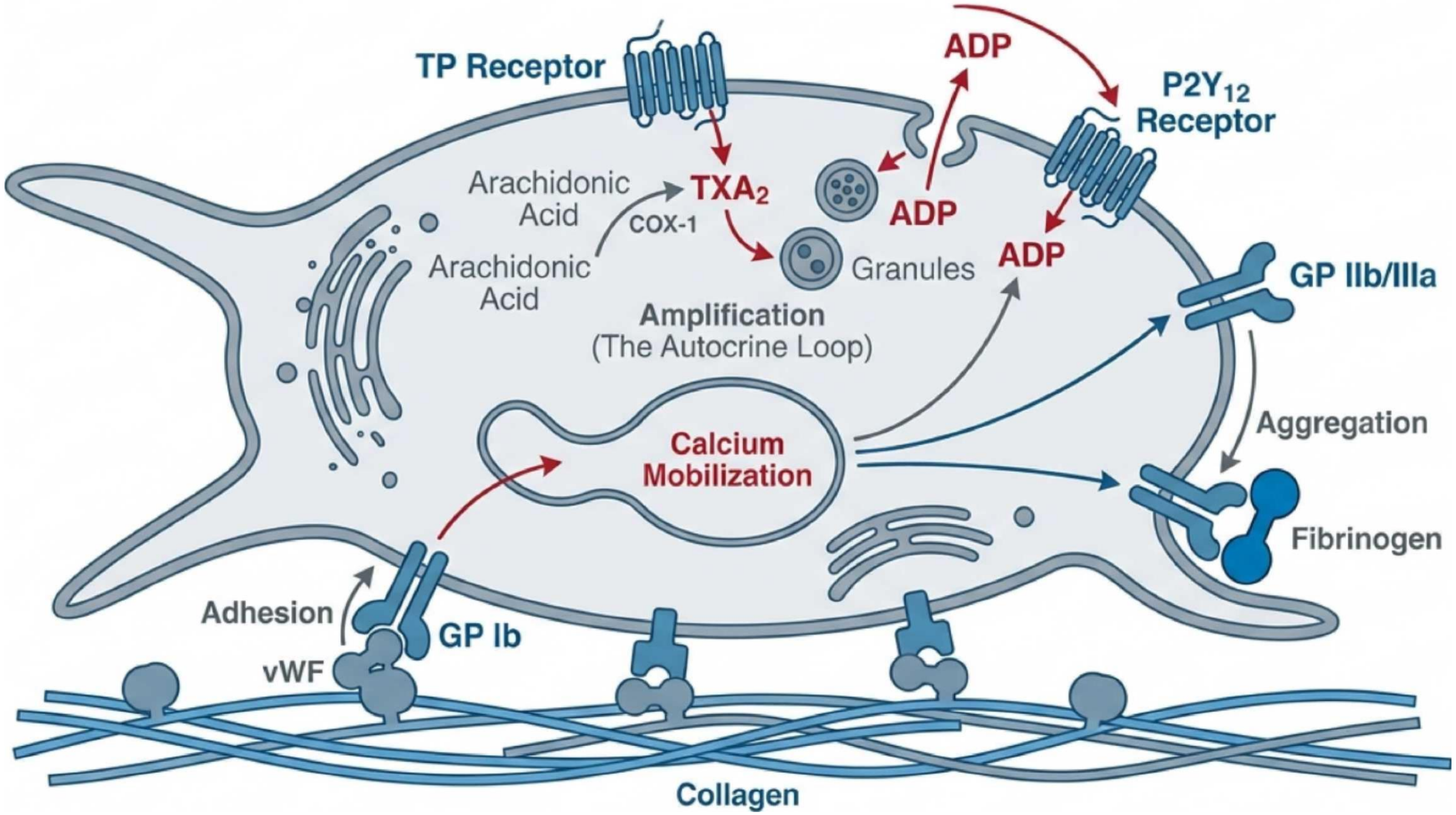

Platelets don't just stick — they **signal, amplify, and stabilize**

Activation = signaling
Aggregation = physical binding (GPIIb/IIIa)

Measuring Function: Light Transmission Aggregometry (LTA)

Resting Platelets (0% Aggregation)

Aggregated Platelets (100% Aggregation)

•Before aggregation → plasma is cloudy → light cannot pass”
•“After aggregation → particles clump → plasma clears → light passes”
•“So ↑ light = ↑ aggregation”

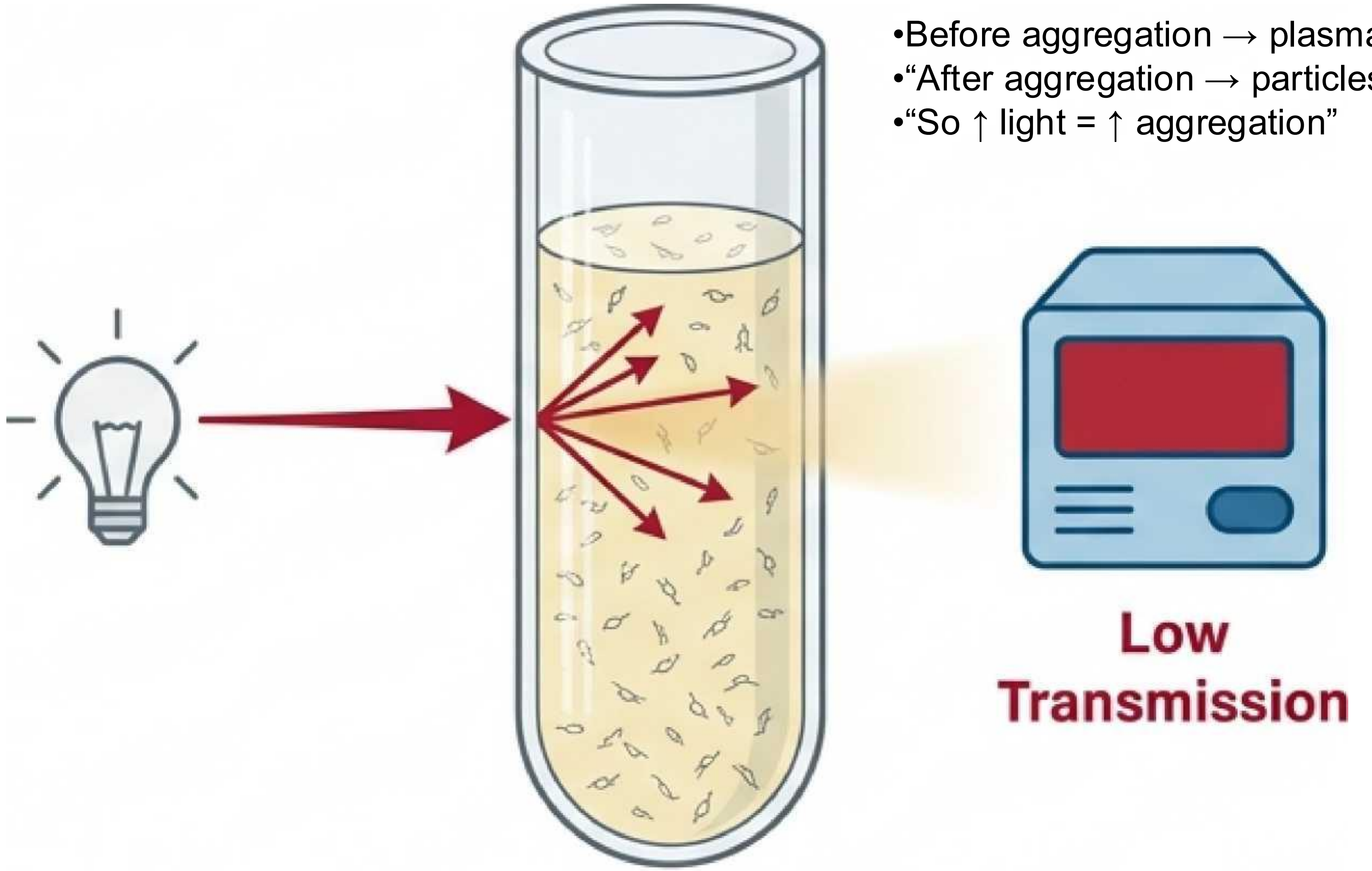

High Turbidity = Light Scatter

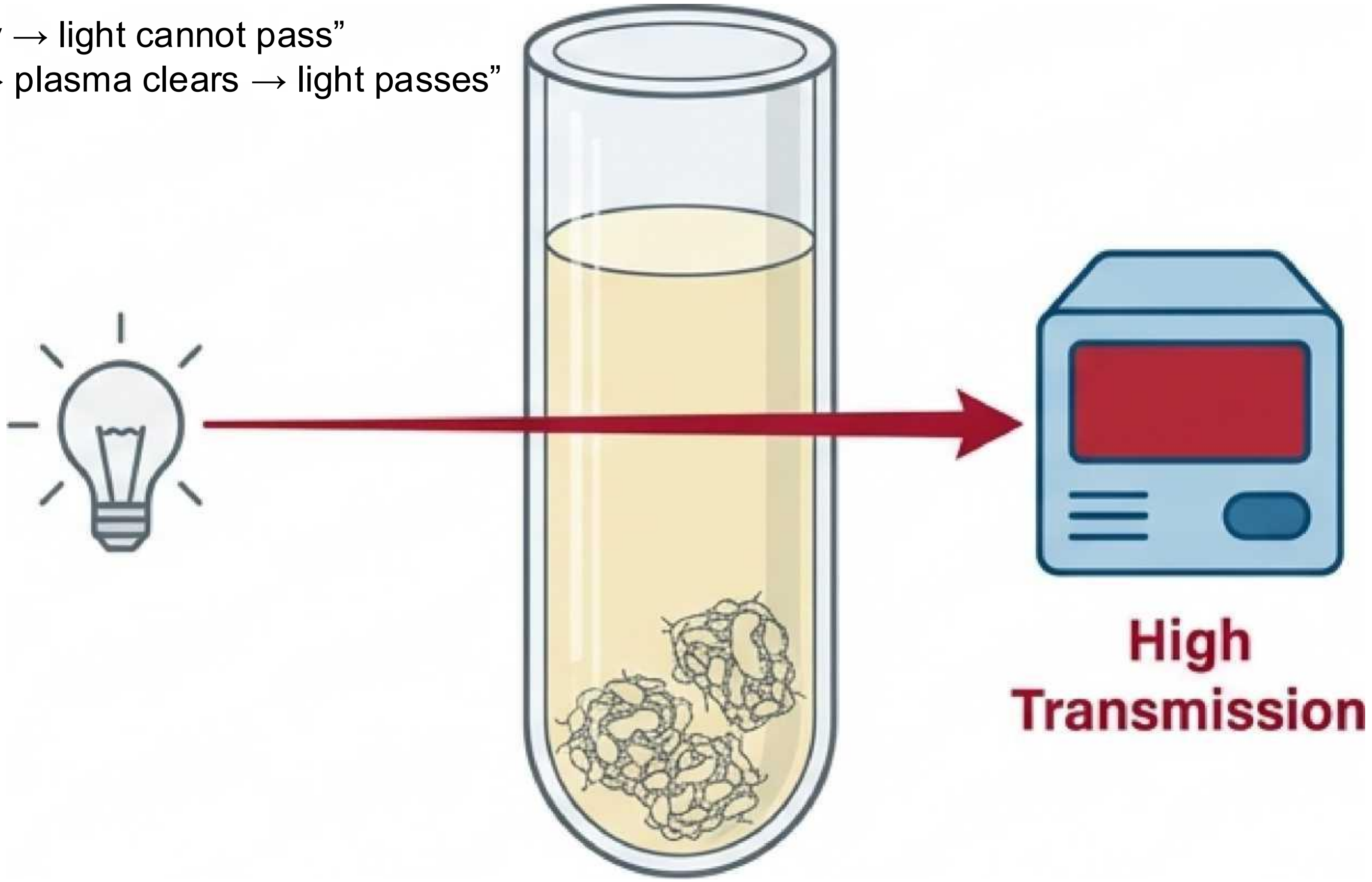

Low Turbidity = High Transmission

The Readout: The Y-Axis of our graph represents % Light Transmission, which is a proxy for the extent of aggregation.

Anatomy of an Aggregation Curve

- **Lag phase** → receptor activation
- **Primary wave** → initial aggregation. direct agonist effect
- **Secondary wave** → amplification phase (ADP + TXA_2)
- **Plateau** → fibrinogen cross-linking (GP IIb/IIIa)

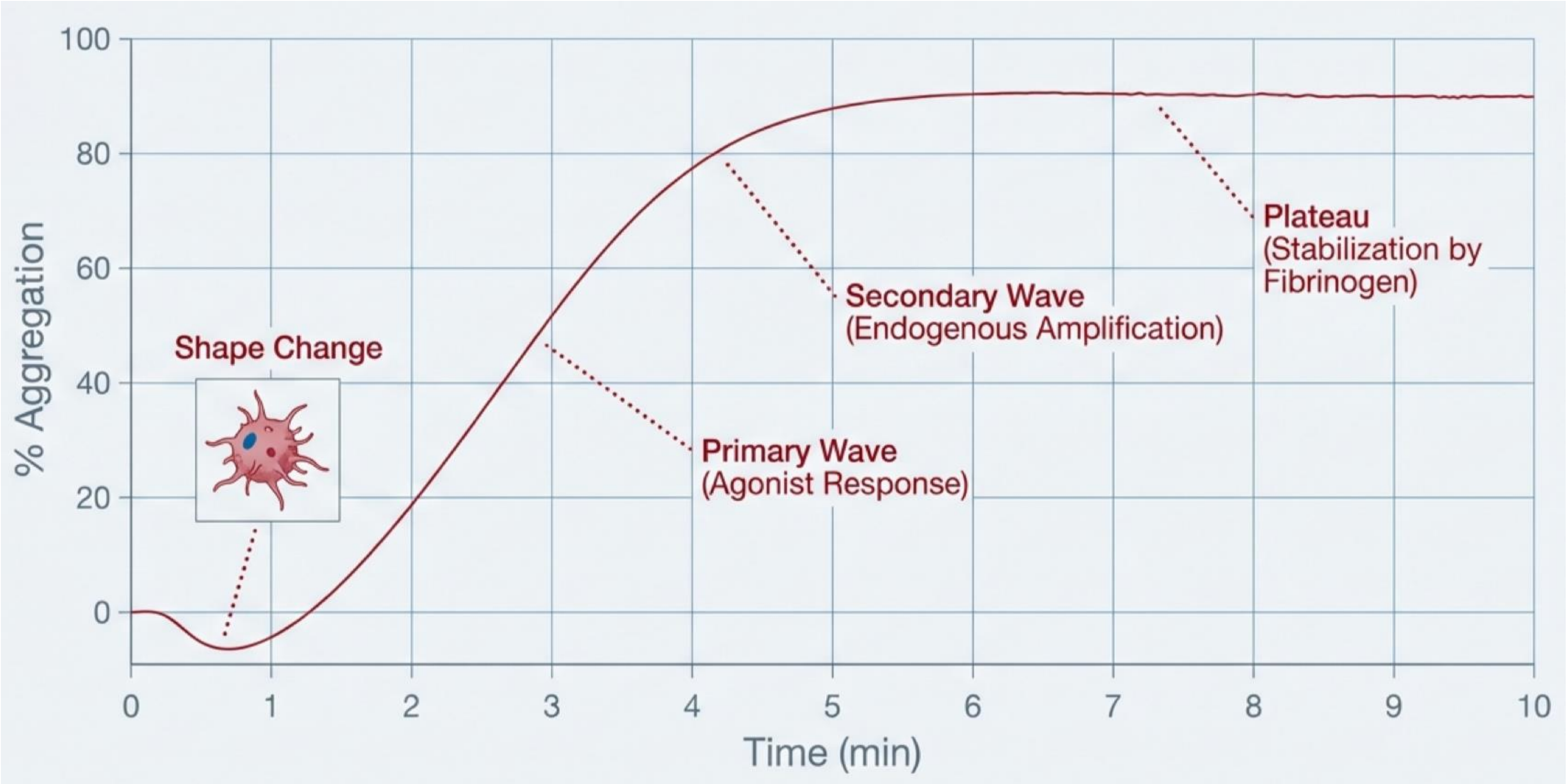

Each drug removes a specific part of the aggregation curve and the missing part tells you the mechanism.

Modulation 1: Cyclooxygenase Inhibition (Aspirin)

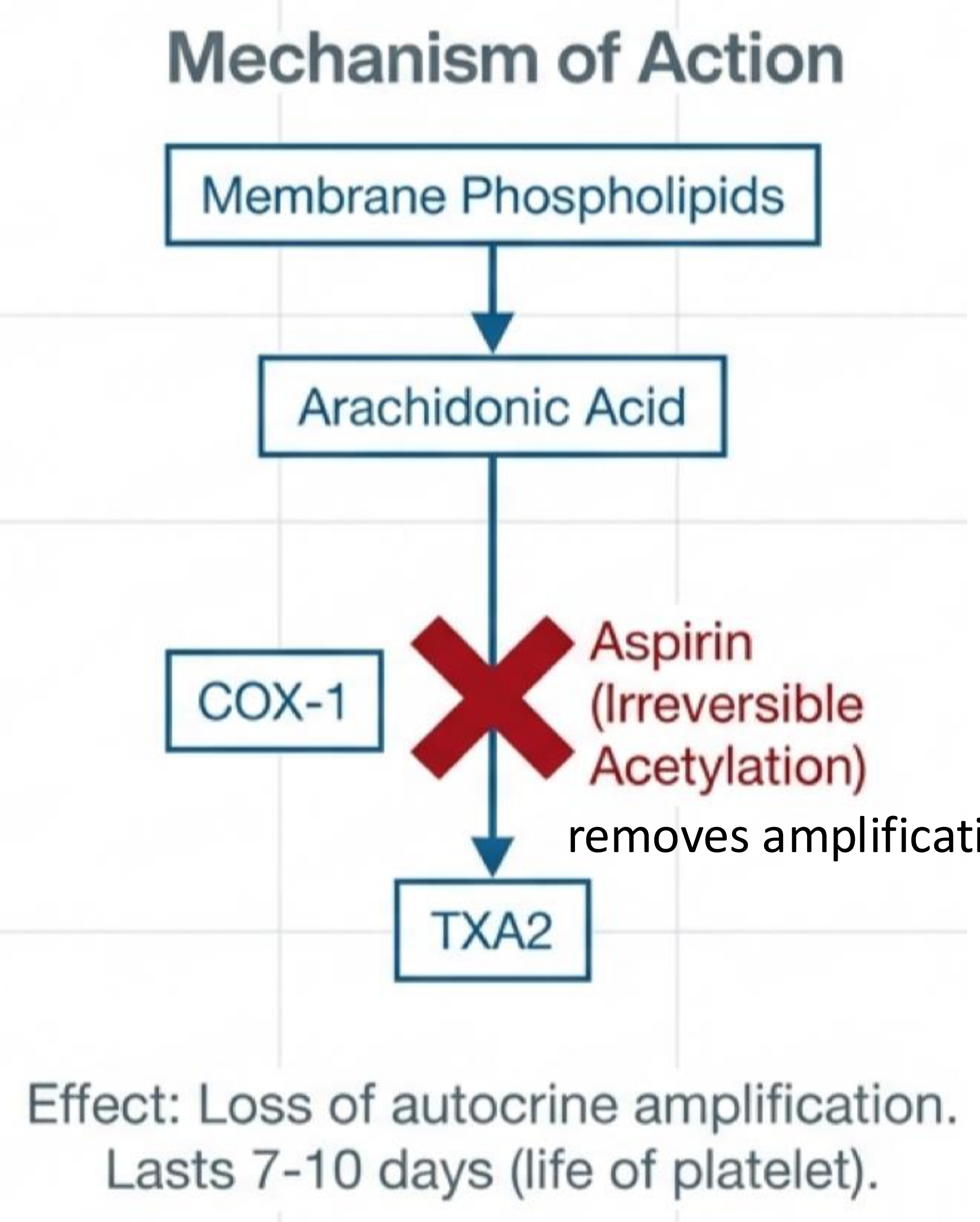

removes amplification

primary wave present, secondary wave weak or absent

Predicted Aggregation Curve

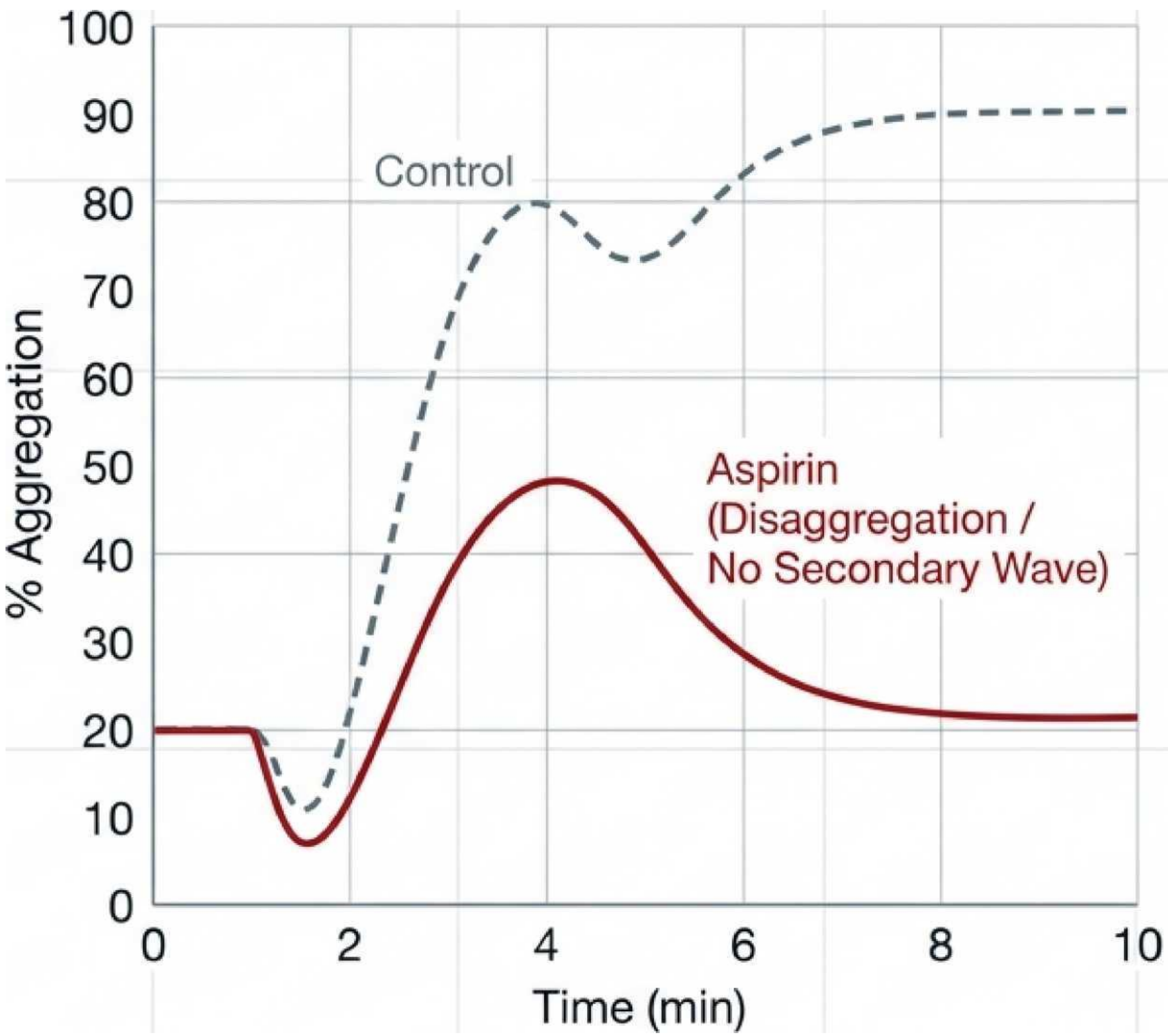

Modulation 2: P2Y12 Receptor Antagonism

Mechanism of Action

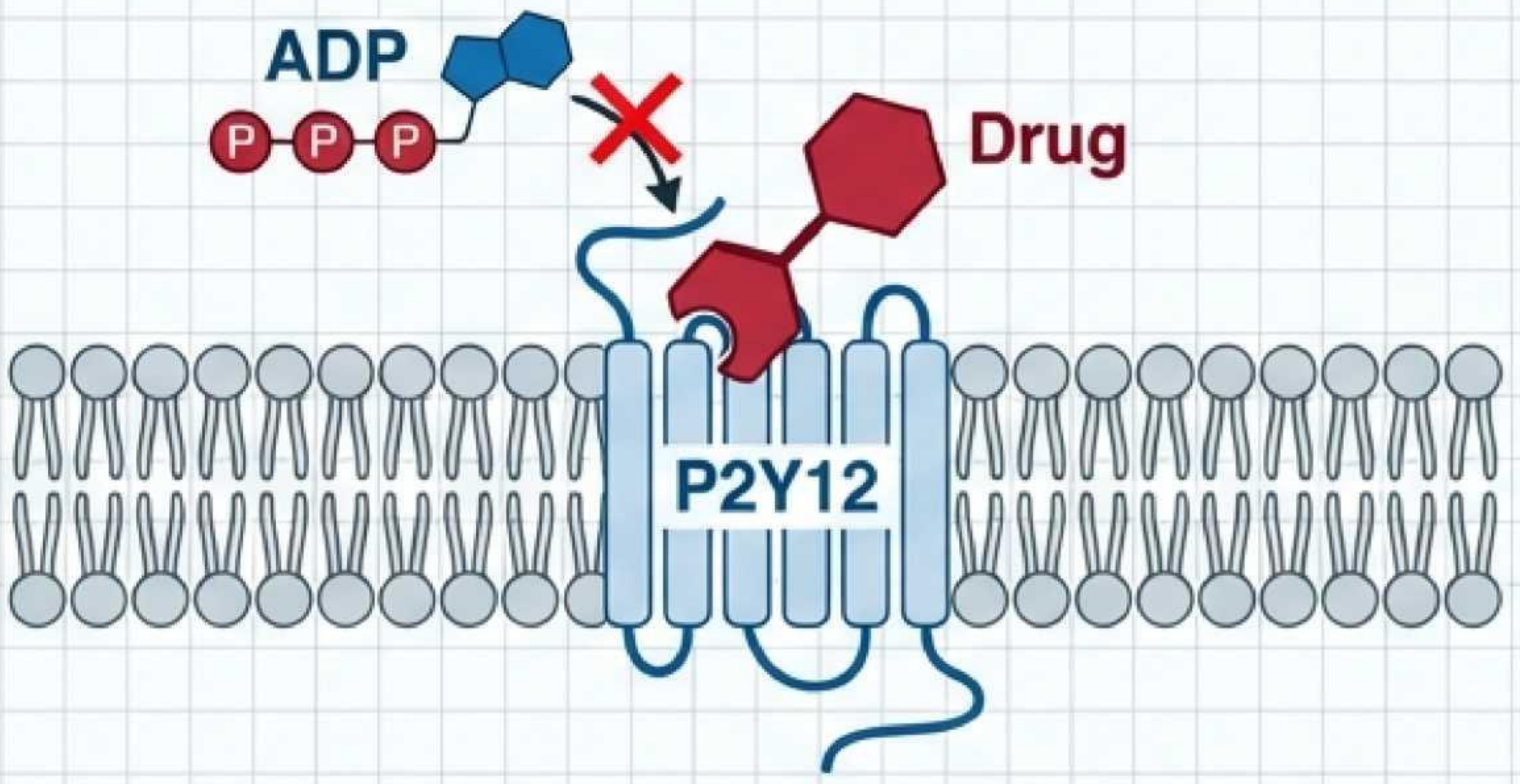

Target: Gi-coupled ADP Receptor.

- **Clopidogrel / Prasugrel:** Irreversible (Prodrugs).
- **Ticagrelor:** Reversible (Allosteric).

Predicted Aggregation Curve

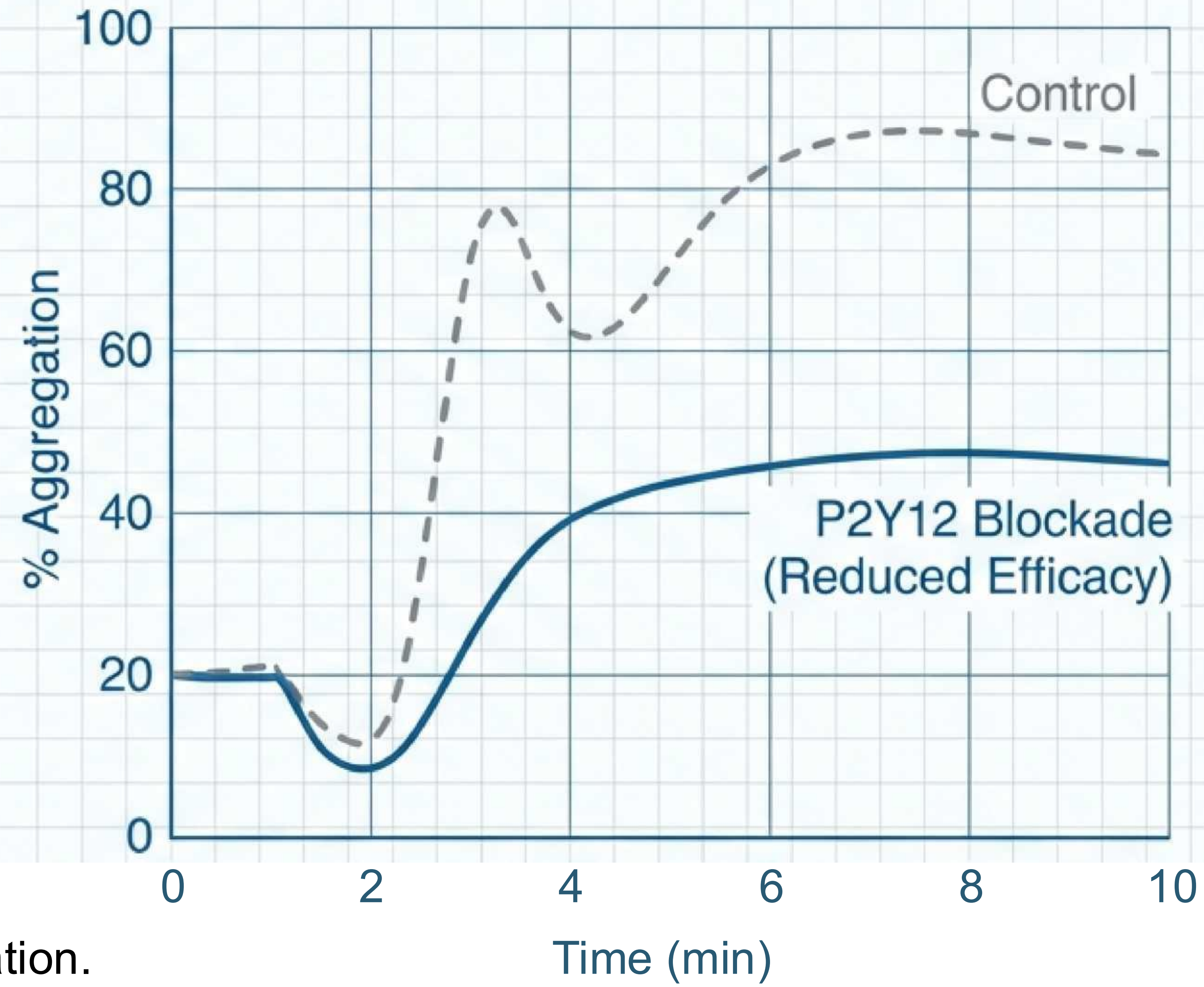

Effect: Prevents ‘sustain’ signal. Reduces GP IIb/IIIa activation.

Modulation 3: The Final Common Pathway (GP IIb/IIIa)

Mechanism of Action

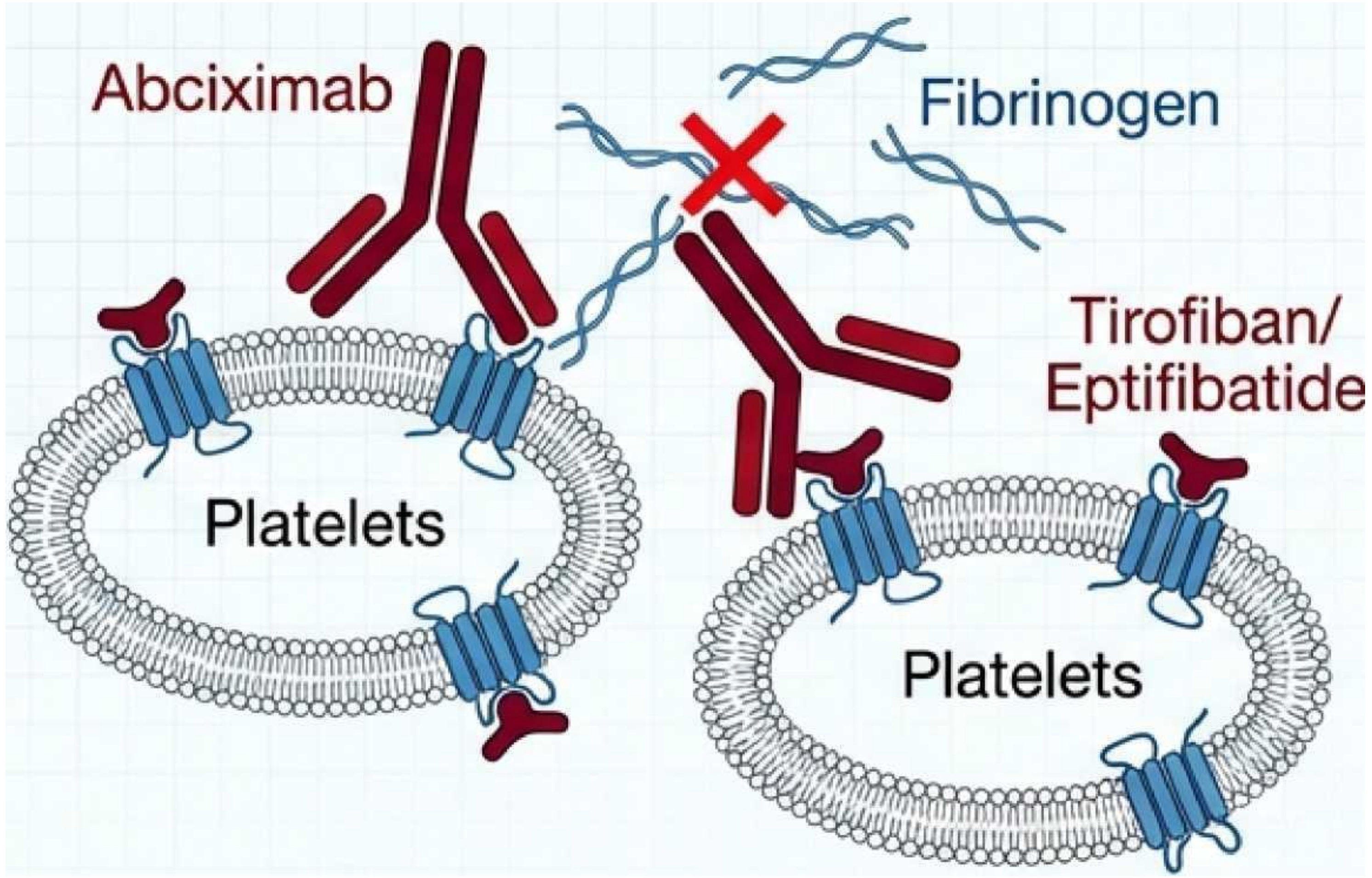

Target: GP IIb/IIIa Integrin.

Agents: Abciximab, Eptifibatide, Tirofiban.

Effect: Steric hindrance of fibrinogen binding.
Mechanical failure of aggregation.

Predicted Aggregation Curve

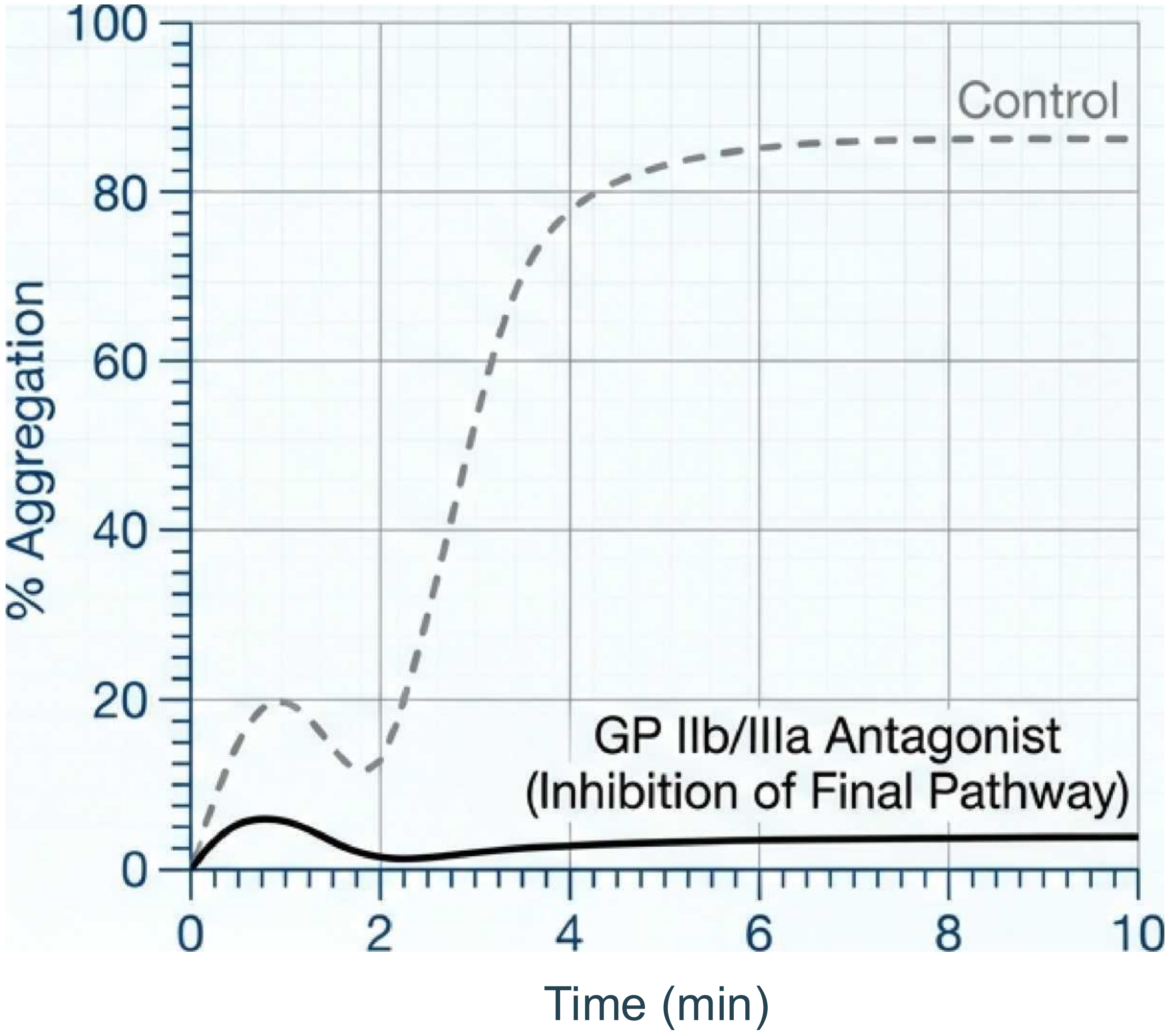

Coagulation Cascade & Anticoagulants

In Silico Pharmacology & Simulation Lab

"From Primary Hemostasis to Secondary Hemostasis"

Platelet aggregation forms the initial unstable plug.
Coagulation stabilizes the plug through fibrin formation.

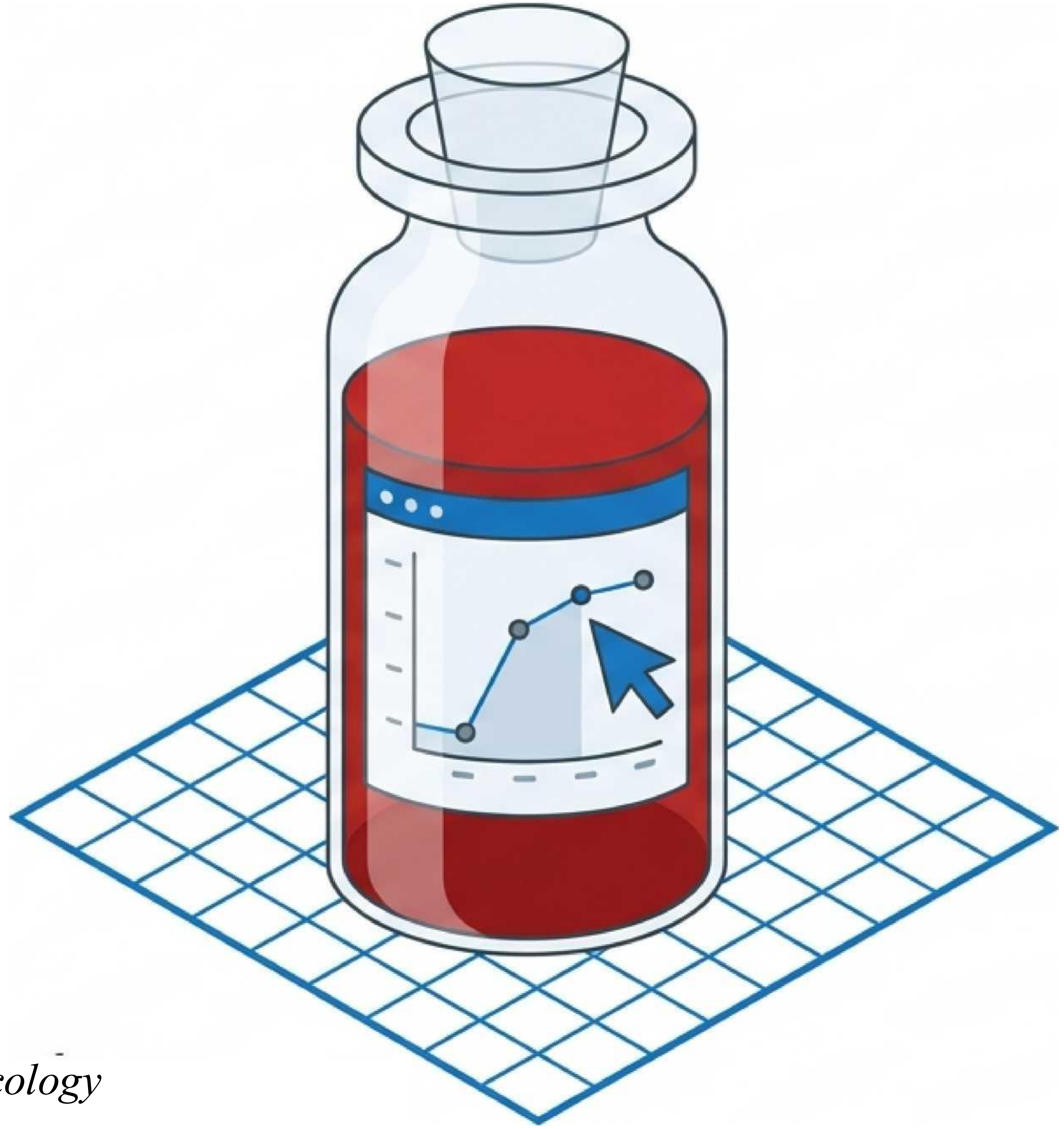

Target Audience: PharmD Year 2 | Focus: Experimental Pharmacology

Session Objectives: The Experimental Logic

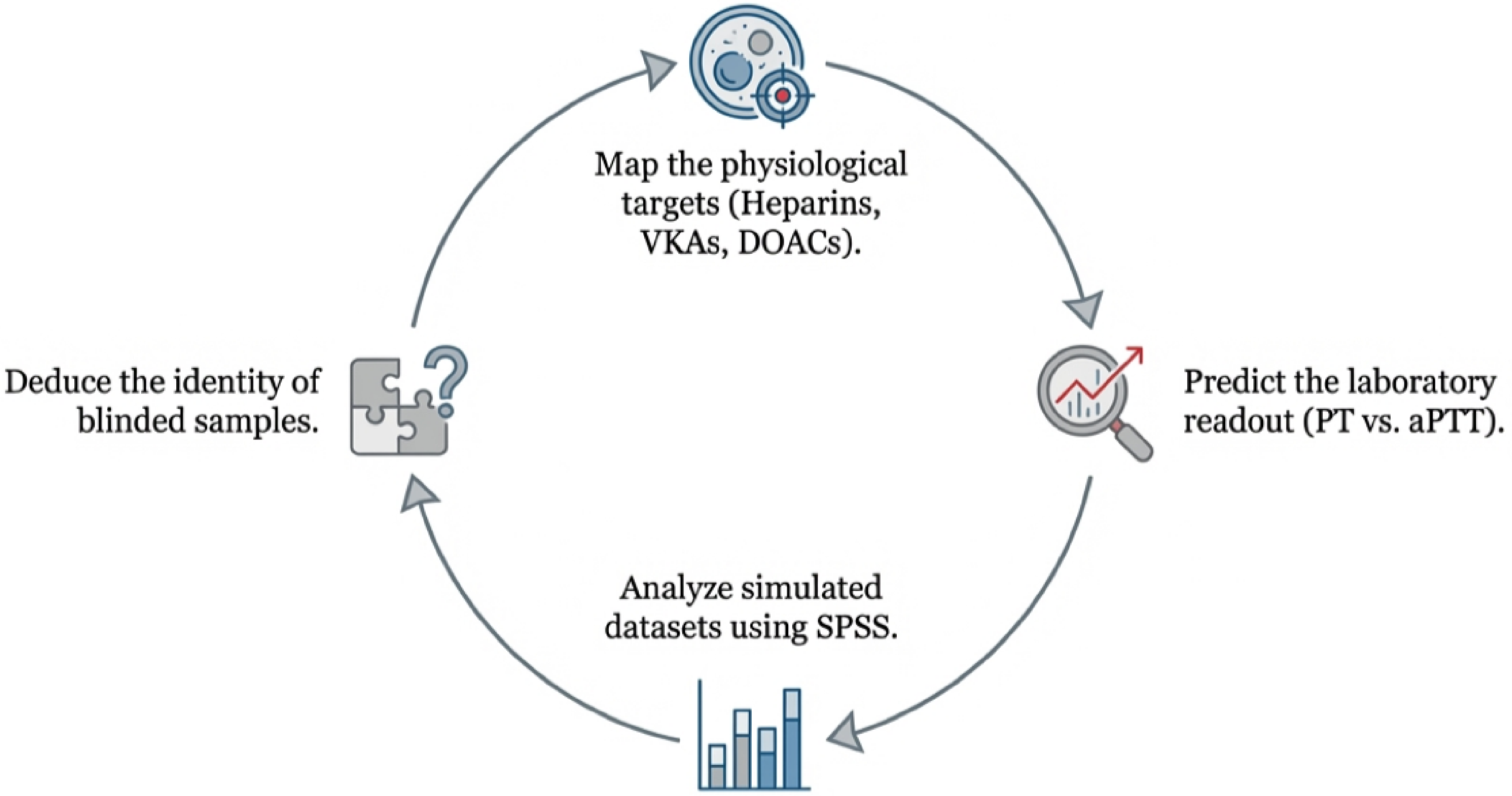

The System: Anatomy of the Coagulation Cascade

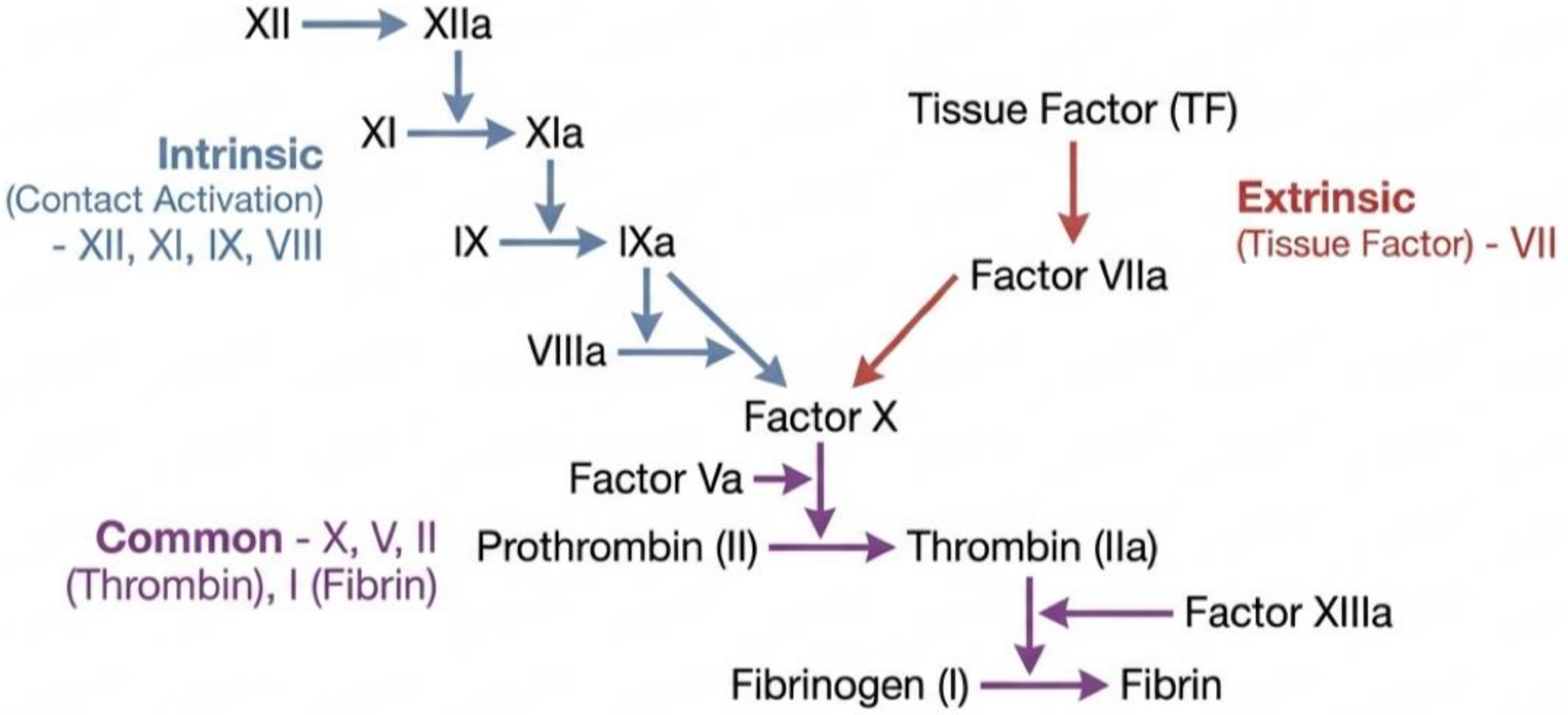

The 'Y' Convergence: Two paths to one outcome (Thrombin generation).
Georgia Pro

The Tools: Measuring the Pathways

Activated Partial Thromboplastin Time (aPTT)

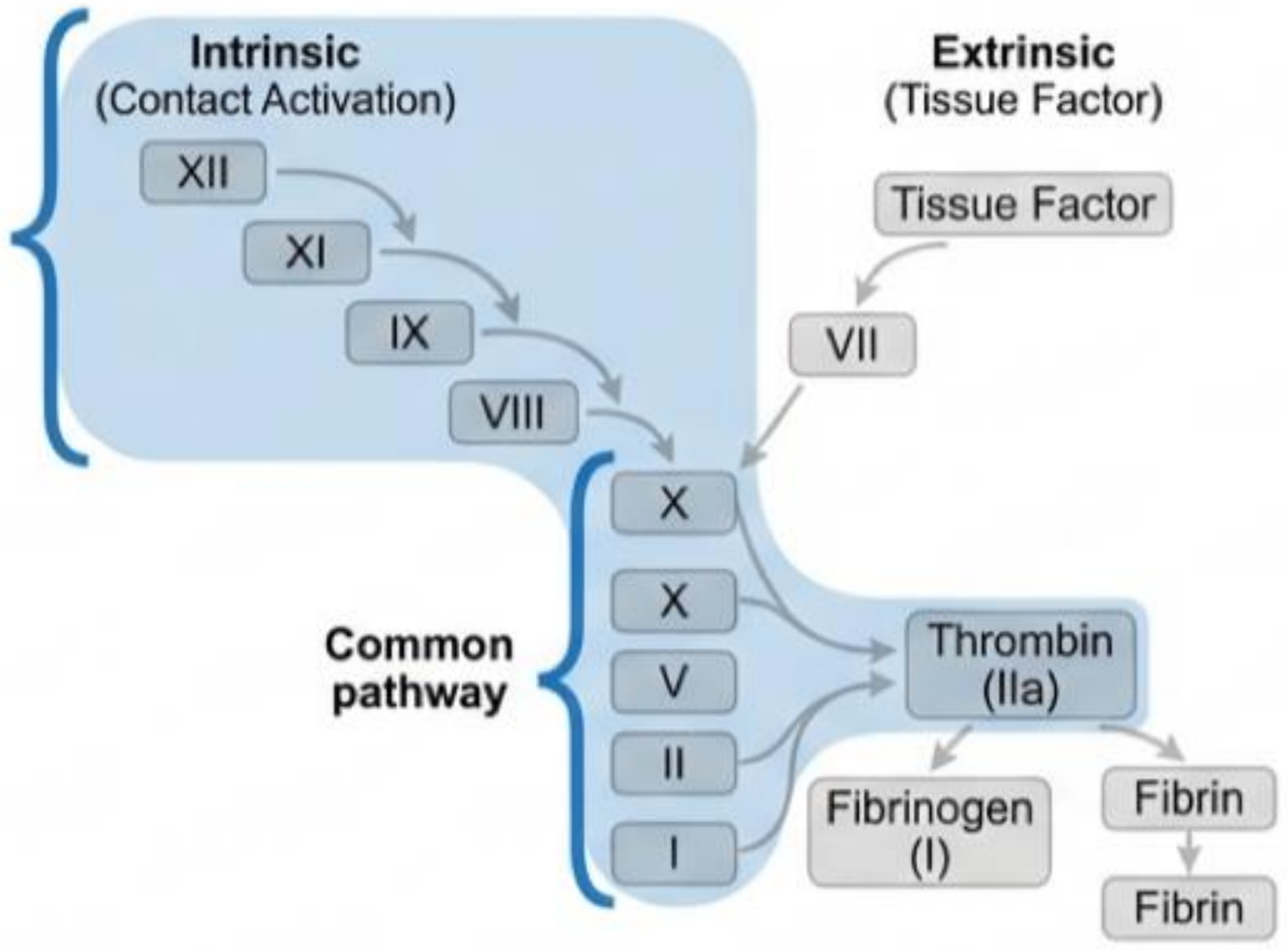

Reagent: Silica/Kaolin + Phospholipids
Measures: XII, XI, IX, VIII, X, V, II, I
Normal Range: ~30–40 seconds

Prothrombin Time (PT)

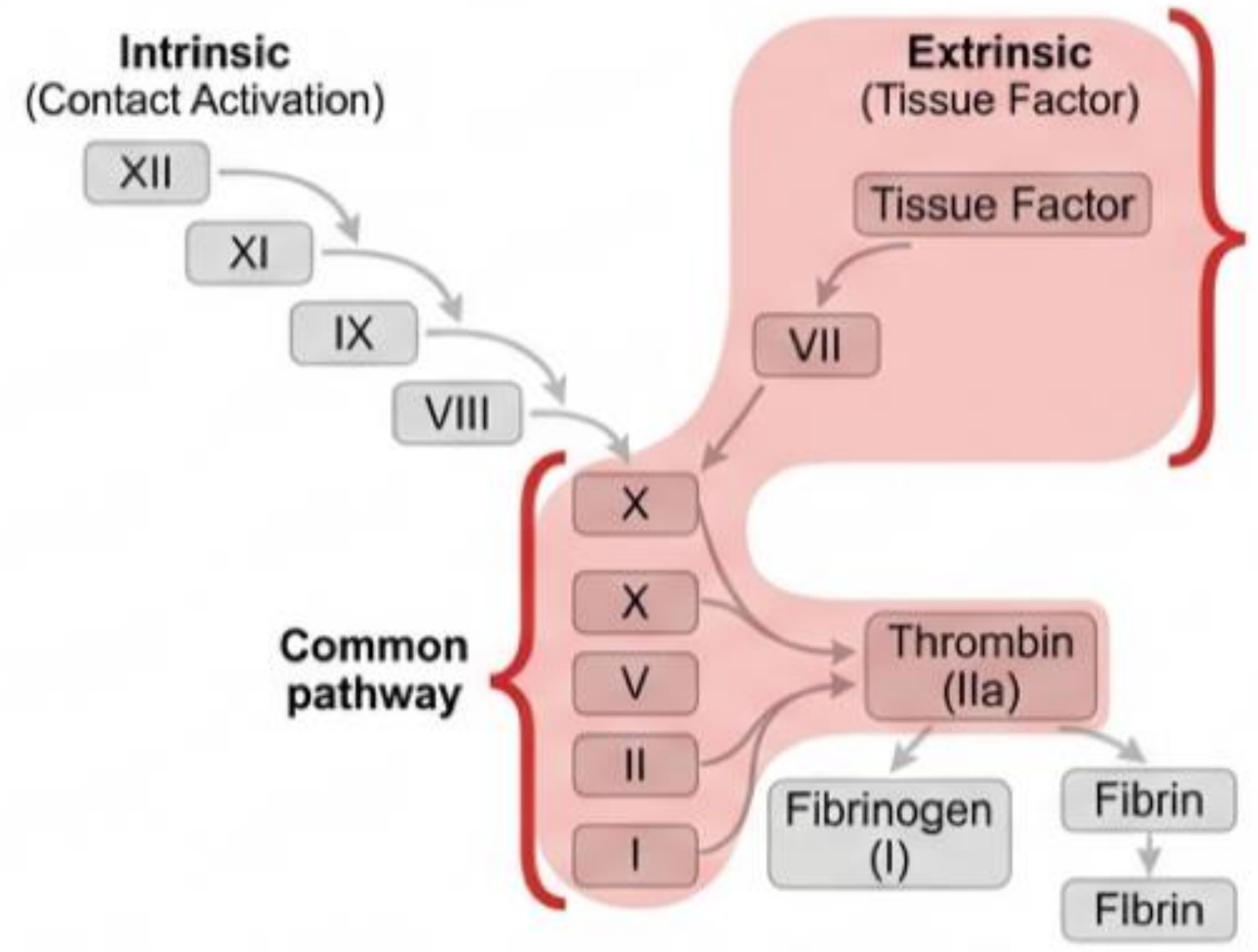

Reagent: Tissue Factor + Calcium
Measures: VII, X, V, II, I
Normal Range: ~12 seconds

The “bottleneck” factor defines sensitivity: Factor VII is unique to the Extrinsic path (PT).

Mechanism I: Indirect Inhibition: Heparins)

A. Unfractionated Heparin (UFH)

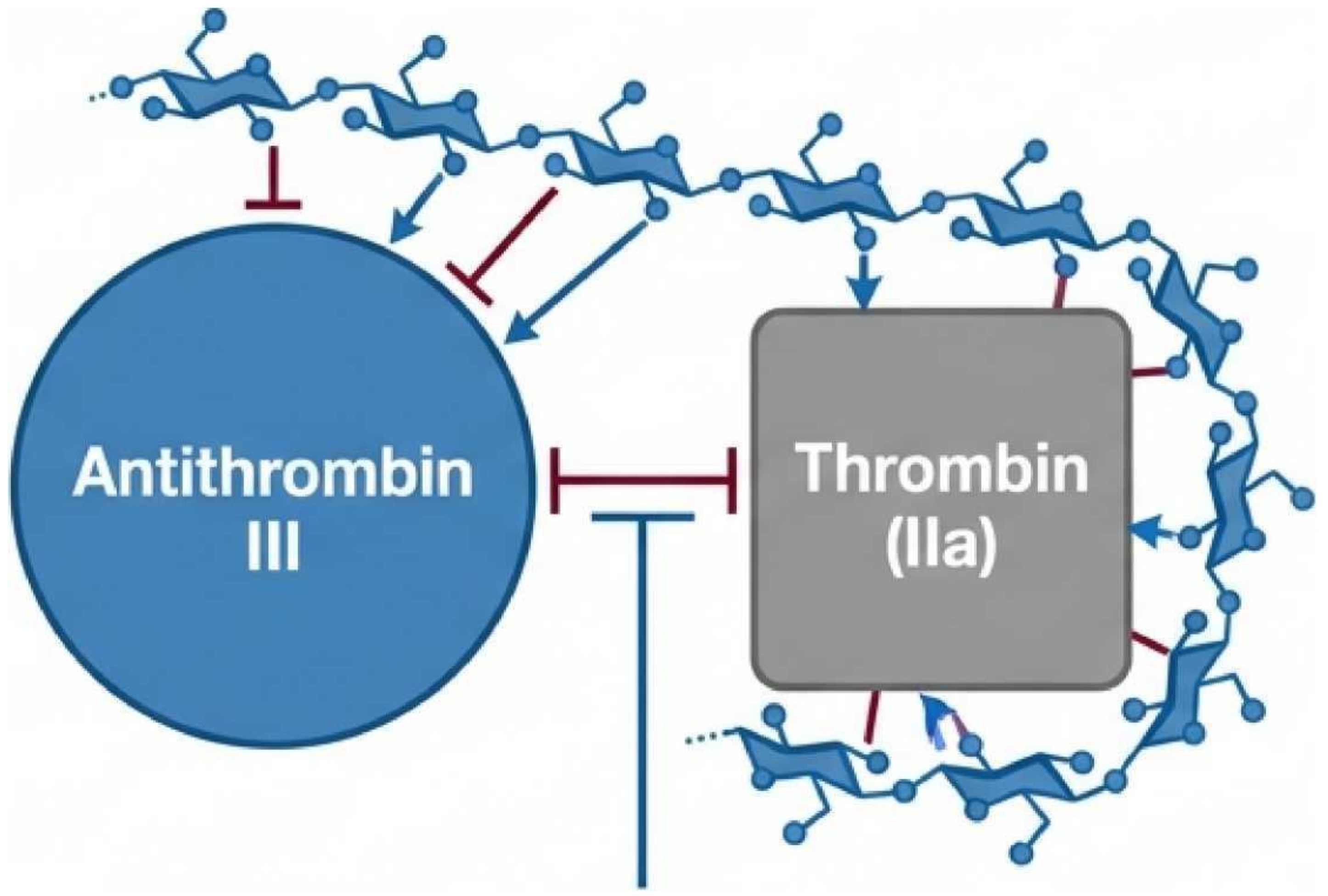

UFH: Bridges AT-III and Thrombin.
Inhibits IIa & Xa.

B. Low Molecular Weight Heparin (LMWH)

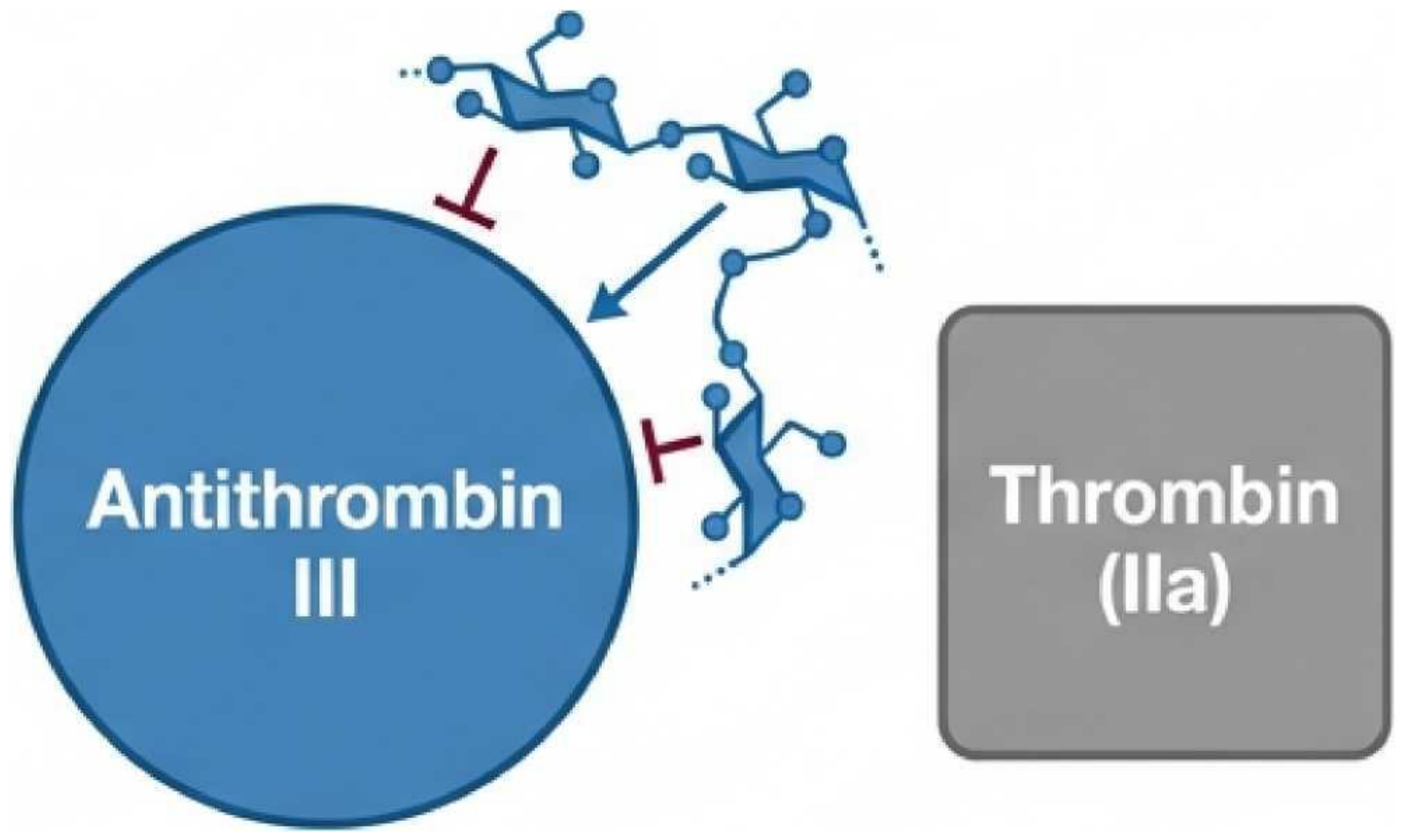

LMWH: Binds AT-III only.
Preferential inhibition of Xa.

Core Mechanism: Potentiation of Antithrombin III (Catalyst)

Mechanism II: Vitamin K Antagonism (Warfarin)

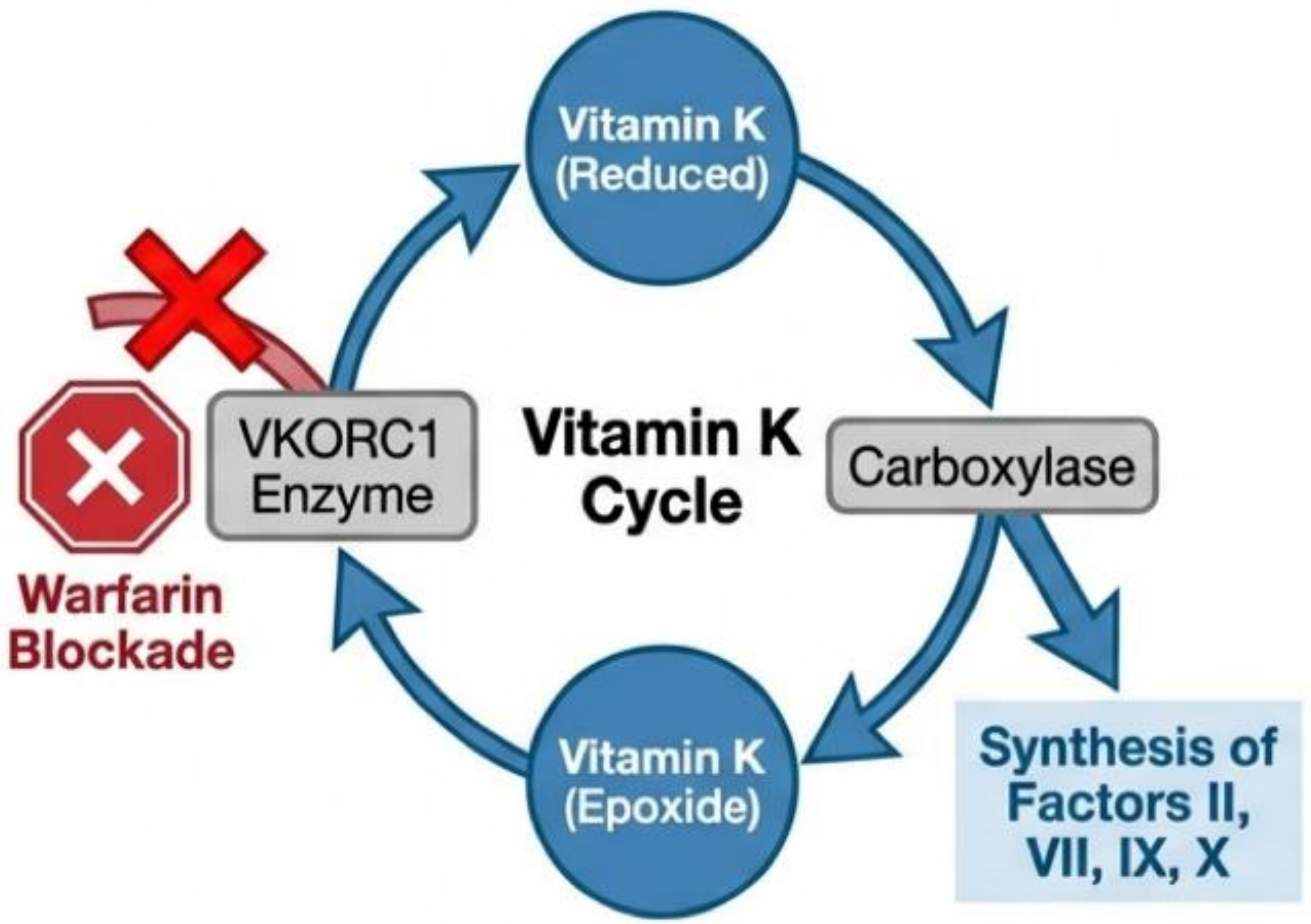

Liver Vitamin K Cycle & Synthesis

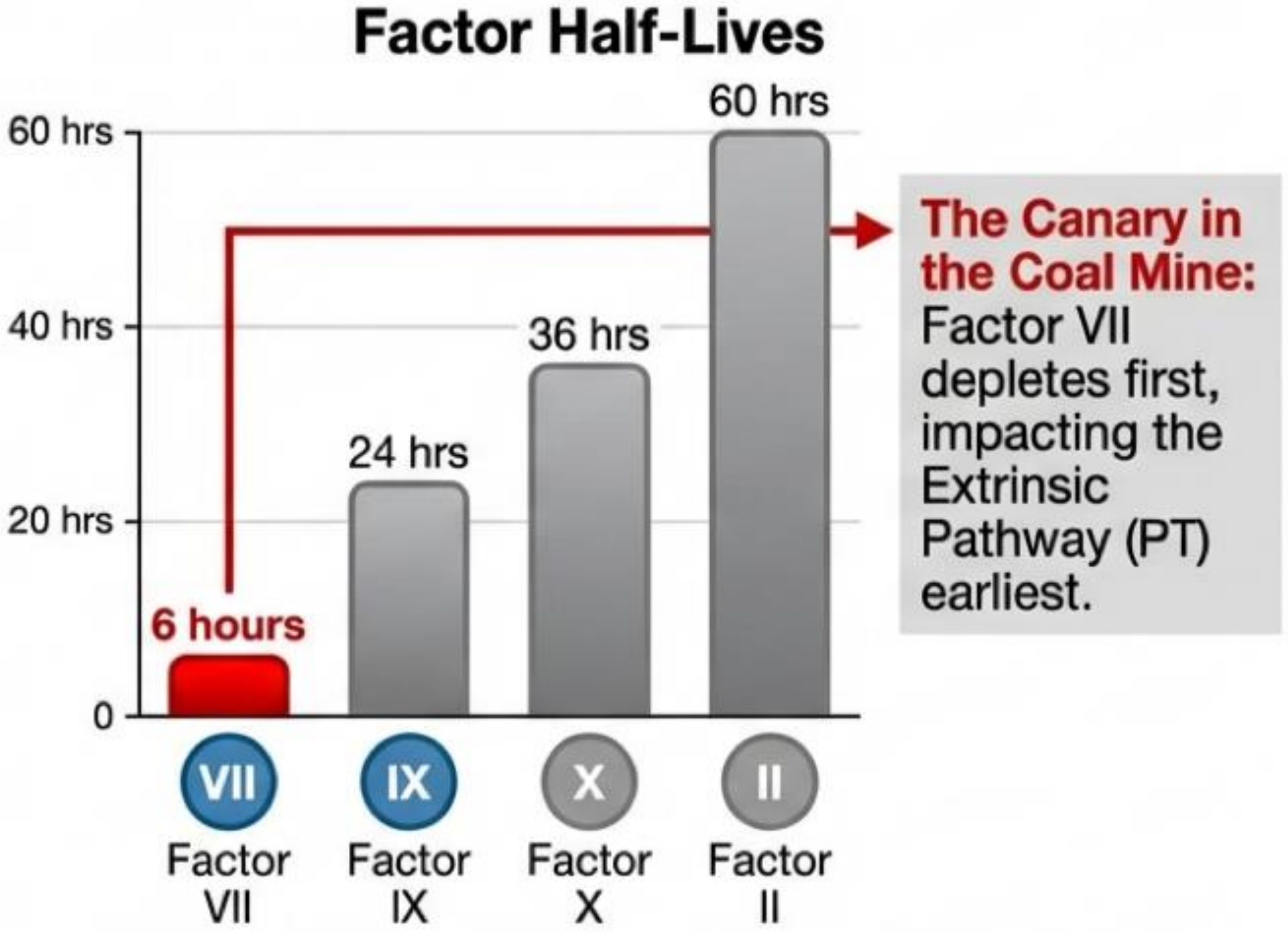

Differential Clotting Factor Half-Lives

Clinical Implication: The Extrinsic Pathway (PT) becomes prolonged earliest due to the rapid depletion of Factor VII, before the Intrinsic/Common pathways are significantly affected.

Mechanism III: Direct Oral Anticoagulants (DOACs)

Direct Thrombin Inhibitors (e.g., Dabigatran)

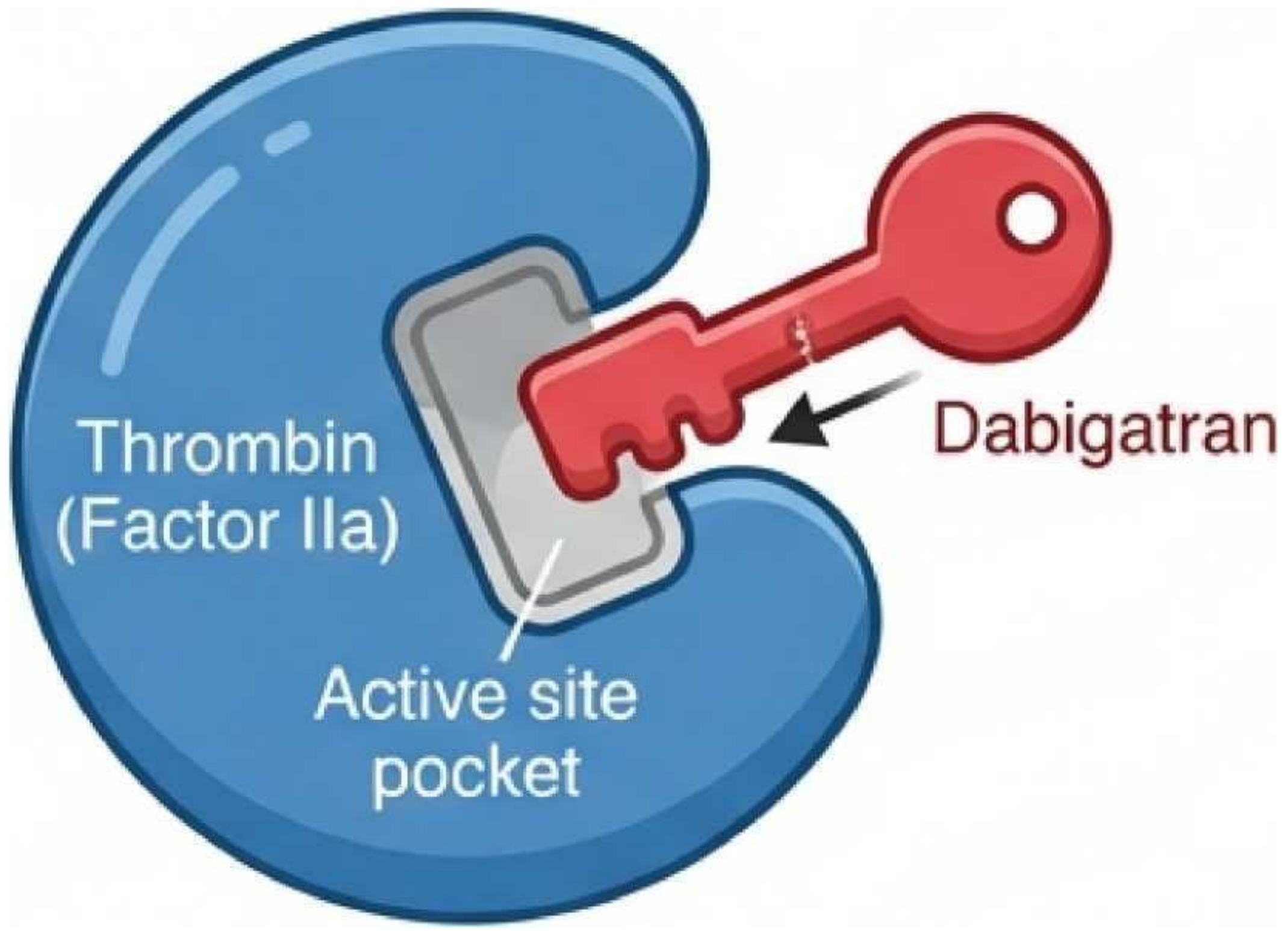

Direct Thrombin Inhibitor.
No cofactor required.

Direct Factor Xa Inhibitors (e.g., Rivaroxaban)

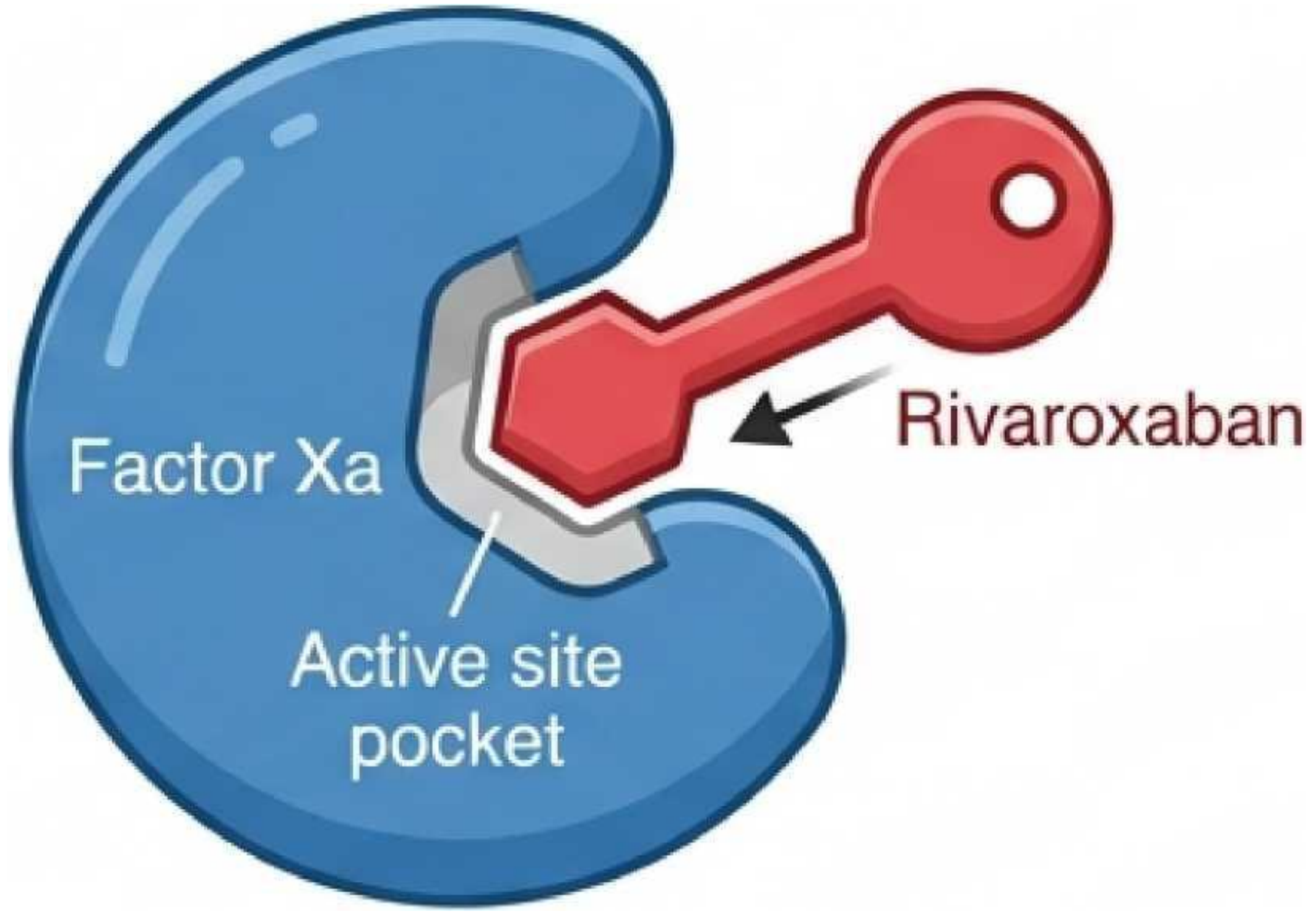

Direct Xa Inhibitor.
Acts at the convergence point.

Lab Effect: Variable. DTIs prolong aPTT > PT. Xa inhibitors affect both, but standard tests are qualitative, not quantitative.

Experimental Logic: Predicting the Shift

Based on the interrupted pathway, predict which coagulation timer will prolong significantly.

Hypothesis Worksheet		
**Drug Class**	**Target Pathway**	**Predicted Lab Shift**
Unfractionated Heparin	Intrinsic + Common (IIa/Xa)	↑↑ aPTT (Major)
Warfarin	Extrinsic (Factor VII - early)	↑↑ PT (Major)
Dabigatran (DTI)	Common (IIa)	↑ aPTT > PT

Synthesis: The ‘Decoder Ring’

Drug Class	Target	Primary Lab Effect	Mechanism
UF Heparin	IIa, Xa	↑↑ aPTT	AT-III Catalyst
LMW Heparin	Xa	↔ / ↑ aPTT	AT-III Catalyst (Xa pref.)
Warfarin	II, VII, IX, X	↑↑ PT (INR)	VKORC1 Inhibitor
Dabigatran	IIa (Direct)	↑ aPTT	Direct Binding
Rivaroxaban	Xa (Direct)	↑ PT & aPTT	Direct Binding

Key Takeaways & Next Steps

1. **Mechanism drives Measurement:** The inhibited factor determines the test (PT vs. aPTT).

2. **Sensitivity:** **Factor VII** is the ‘canary’ for **Warfarin (PT)**; **Thrombin** drives **Heparin monitoring** (aPTT).

3. **Data Limitations**: **DOACs** affect screens but require **specific assays** for quantification.

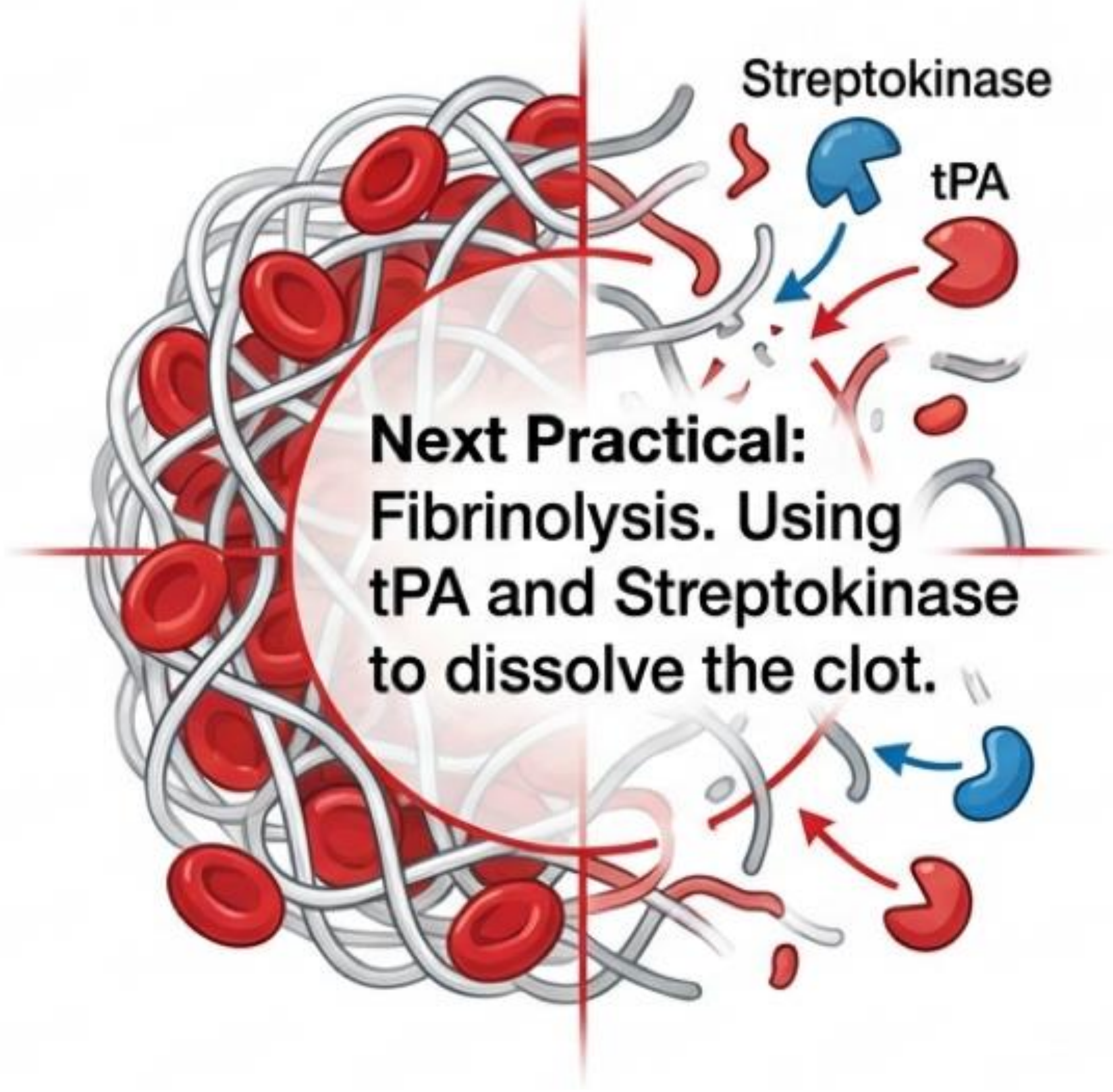

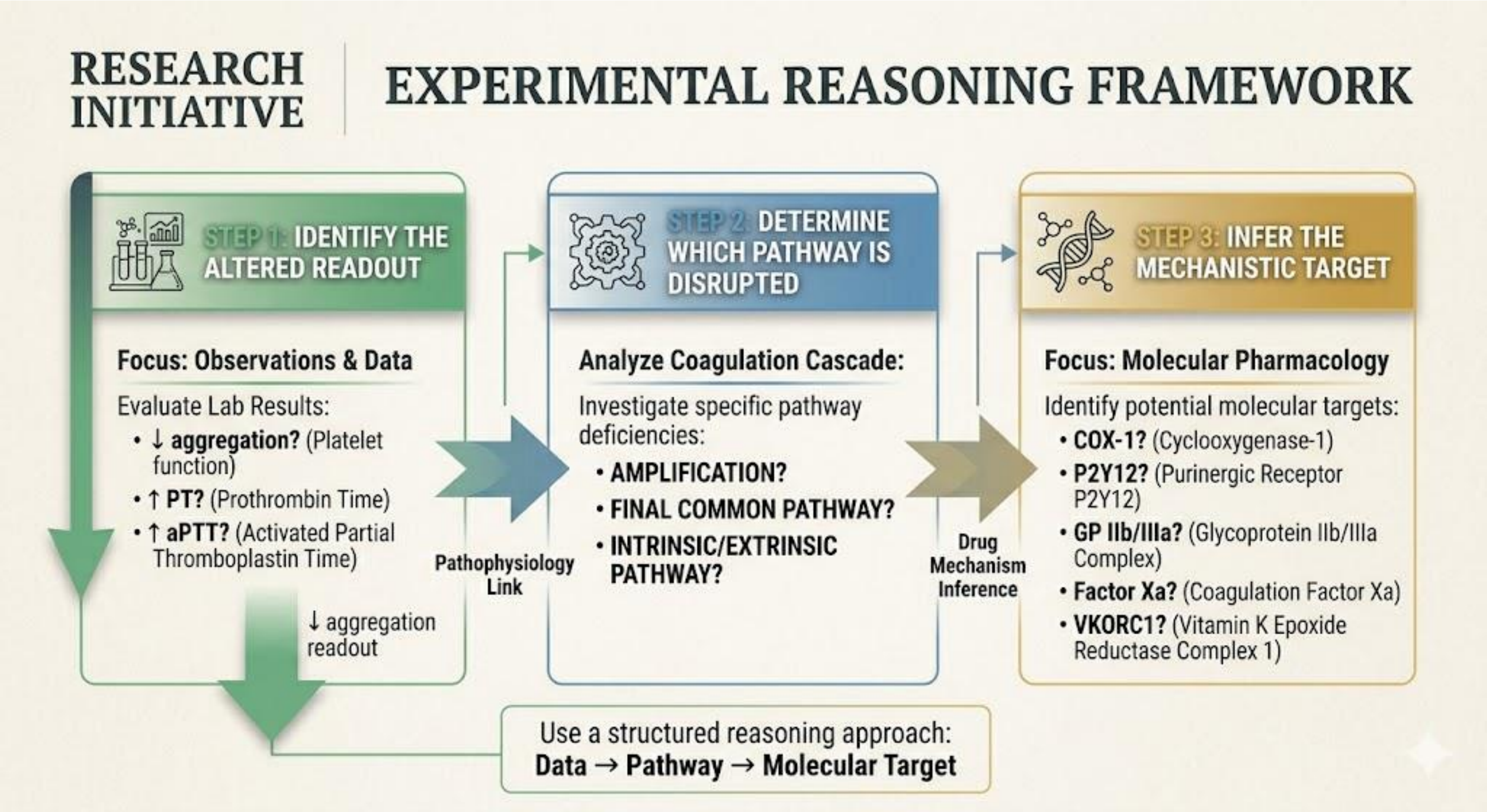
RESEARCH INITIATIVE
EXPERIMENTAL REASONING FRAMEWORK
STEP 1: IDENTIFY THE ALTERED READOUT
Focus: Observations & Data
Evaluate Lab Results:
• ↓ aggregation? (Platelet function)
• ↑ PT? (Prothrombin Time)
• ↑ aPTT? (Activated Partial Thromboplastin Time)
↓ aggregation readout
Pathophysiology Link
STEP 2: DETERMINE WHICH PATHWAY IS DISRUPTED
Analyze Coagulation Cascade:
Investigate specific pathway deficiencies:
• AMPLIFICATION?
• FINAL COMMON PATHWAY?
• INTRINSIC/EXTRINSIC PATHWAY?
Drug Mechanism Inference
STEP 3: INFER THE MECHANISTIC TARGET
Focus: Molecular Pharmacology
Identify potential molecular targets:
• COX-1? (Cyclooxygenase-1)
• P2Y12? (Purinergic Receptor P2Y12)
• GP IIb/IIIa? (Glycoprotein IIb/IIIa Complex)
• Factor Xa? (Coagulation Factor Xa)
• VKORC1? (Vitamin K Epoxide Reductase Complex 1)
Use a structured reasoning approach:
Data → Pathway → Molecular Target
