# Supplementary material for: Evaluation of a multi-component AI-guided mechanistic reasoning practical in experimental pharmacology: a single-group pre-post study
Source: Front Pharmacol. 2026 Sep 21;17:1937176. doi: 10.3389/fphar.2026.1937176 (PMC13634817; doi:10.3389/fphar.2026.1937176)
Supplement: Supplementary file 3 [file DataSheet3.pdf]

Step 3: AI-Supported Experimental Reasoning PracticalExperimental Pharmacology

Student Practical Worksheet
You will analyze simulated experimental pharmacology datasets involving:· - Platelet aggregation curves - Antiplatelet drug mechanisms - PT/aPTT coagulation profiles - Anticoagulant mechanisms
Your task is to: 1. Identify the major experimental change 2. Determine the affected physiological pathway 3. Infer the likely drug mechanism or drug class4. Use AI only as a guided reasoning assistant You are NOT allowed to ask AI directly for the final answer

AI Prompt Guide/Approved AI Prompt

Students may copy and use the following prompt:
I am a second-year PharmD student analyzing a simulated experimental pharmacology case. I will describe the observed findings from a platelet aggregation graph or coagulation dataset. Do NOT give me the final answer directly. Instead:

1. Ask step-by-step reasoning questions
2. Help me identify what changed in the experimental readout
3. Help me determine which pathway is affected
4. Help me connect the pathway to the likely drug mechanism
5. Guide my reasoning without directly revealing the final answer

Examples of Appropriate AI Use
Appropriate
• "What does loss of the secondary wave usually suggest?"• "Which coagulation pathway mainly affects aPTT?"
• "What is the difference between amplification failure and final aggregation failure?" • "How does PT differ from aPTT mechanistically?"
Inappropriate
• "What is the answer?"
• "Identify the drug directly."
• "Solve this worksheet for me." • "Which option should I choose?"

1

Anonymous Study ID

SECTION A: Platelet Aggregation Cases

Platelet Case 1 – Drug A

Simulated Aggregation Curve

SECTION A: Platelet Aggregation Cases

2

Platelet Case 1 – Drug A Structured Student Analysis

Observed Findings

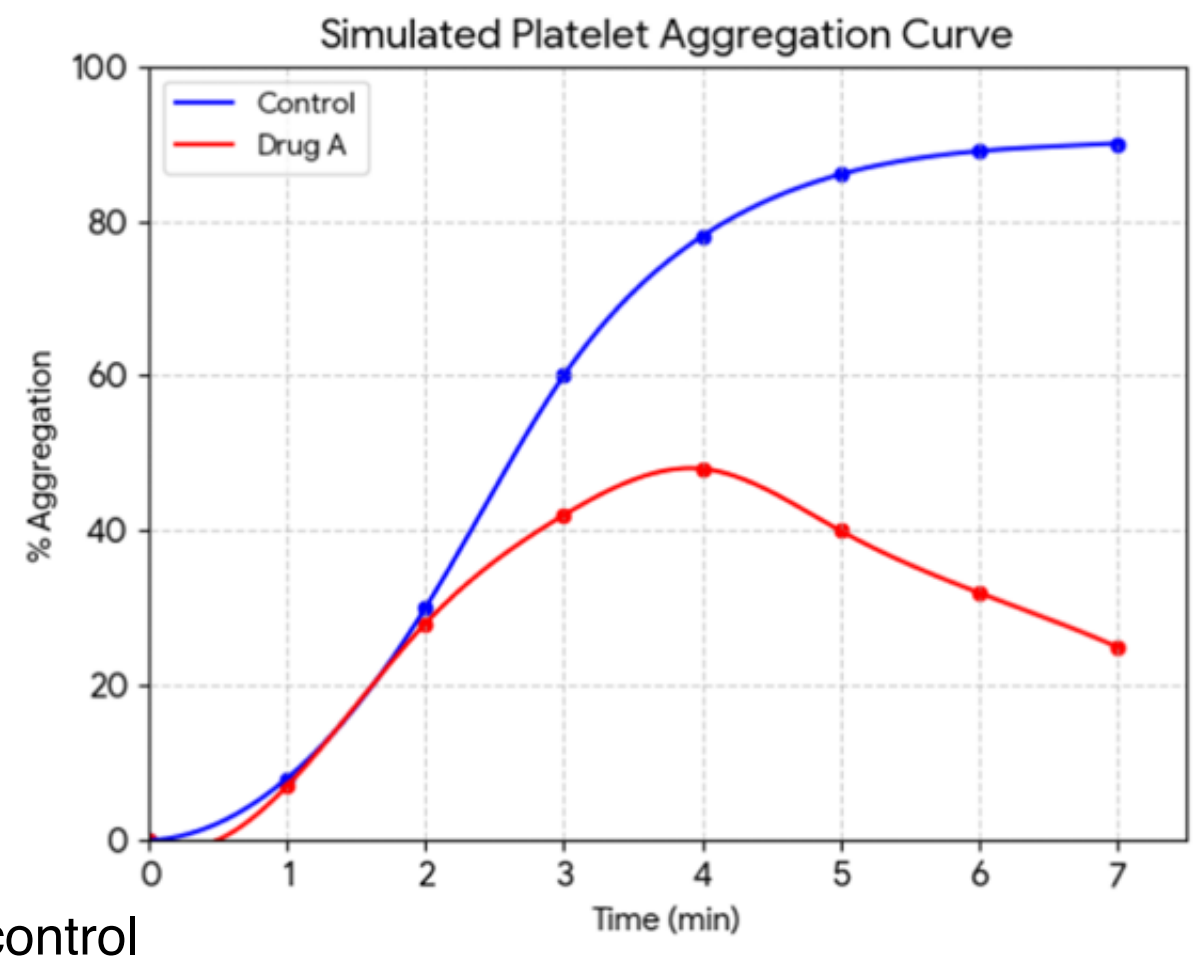

normal lag phase before aggregation begins
preserved initial (primary) aggregation response
reduced sustained aggregation compared with control
aggregation becomes unstable after the initial rise
final aggregation level remains lower than control

3

Q1. What is the major abnormality in Drug A? * (1 Point)

- ◯ A. Delayed lag phase only
- ◯ B. Loss of secondary wave/amplification
- ◯ C. Complete absence of aggregation
- ◯ D. Increased maximal aggregation

4

Q2. Which pathway is MOST likely affected? * (1 Point)

- A. COX-1/TXA2 amplification pathway
- B. GP IIb/IIIa fibrinogen binding
- C. Factor Xa pathway
- D. Extrinsic coagulation pathway

5

Q3. Which drug class is MOST likely represented? * (1 Point)

- A. Aspirin-like
- B. P2Y12 antagonist
- C. GP IIb/IIIa antagonist
- D. Direct thrombin inhibitor

6

Q4. Confidence score *

- ◯ 1 = Not confident
- ◯ 2 = Slightly confident
- ◯ 3 = Moderately confident
- ◯ 4 = Confident
- ◯ 5 = Very confident

7

Q5. Did AI help your reasoning? *

- ◯ Yes
- ◯ No

8

Platelet Case 2 – Drug B

Observed Findings

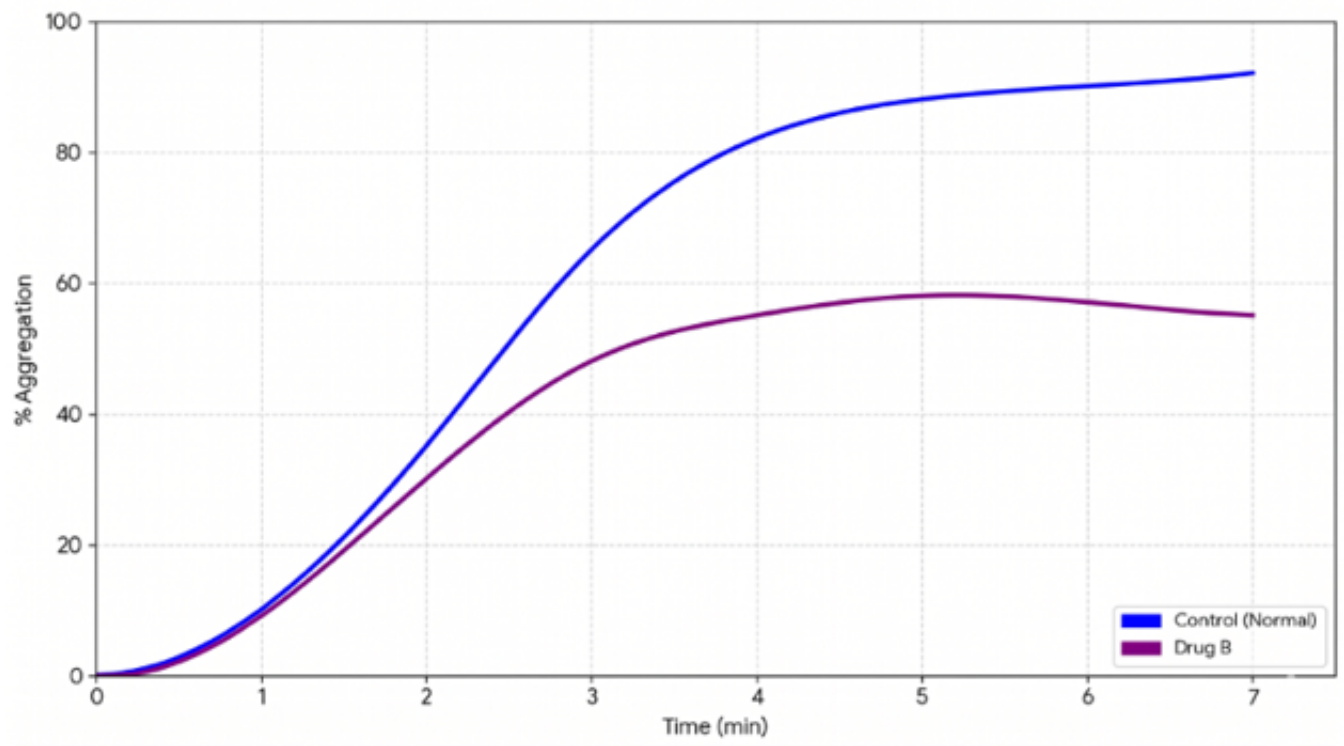

normal lag phase before aggregation begins
preserved initial aggregation response
sustained aggregation is weaker than control
final plateau is reduced compared with control
aggregation remains present but does not reach control levels

9

Q1. What is the major abnormality in Drug B? * (1 Point)

- ◯ Delayed lag phase only
- ◯ Reduced sustained aggregation/amplification
- ◯ Complete absence of aggregation
- ◯ Excessive aggregation

10

Q2. Which pathway is MOST likely affected? * (1 Point)

- A. P2Y12-mediated ADP amplification pathway
- B. GP IIb/IIIa final aggregation pathway
- C. Factor Xa pathway
- D. Extrinsic coagulation pathway

11

Q3. Which drug class is MOST likely represented? * (1 Point)

- A. P2Y12 antagonist
- B. Aspirin-like drug
- C. Warfarin-like drug
- D. GP IIb/IIIa antagonist

12

Q4. Confidence score. *

- ◯ 1 = Not confident
- ◯ 2 = Slightly confident
- ◯ 3 = Moderately confident
- ◯ 4 = Confident
- ◯ 5 = Very confident

13

Q5. Did AI help your reasoning? *

- ◯ Yes
- ◯ No

14

Platelet Case 3 – Drug C

Observed Findings

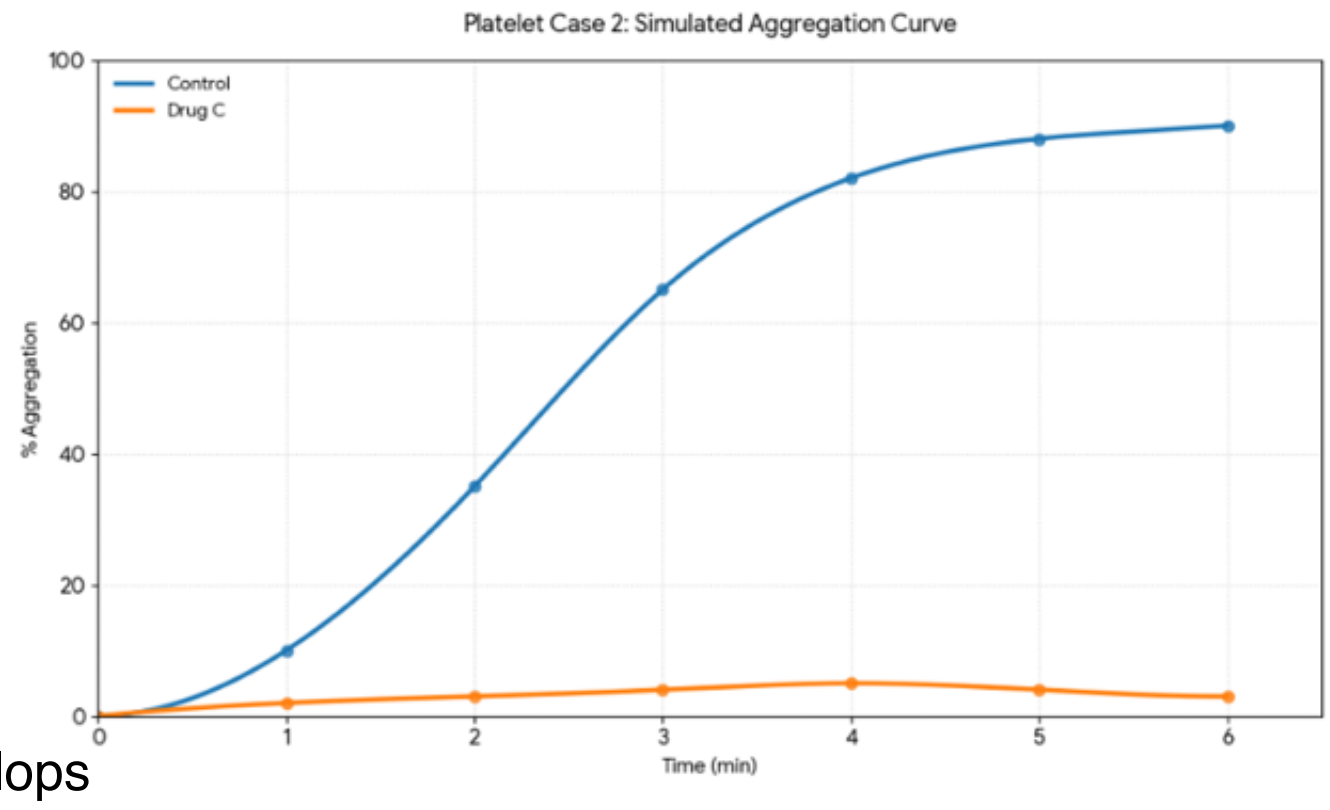

minimal aggregation after stimulation
very small change compared with baseline
no meaningful sustained aggregation develops
response remains markedly lower than control throughout
aggregation is nearly absent regardless of stimulation

15

Q1. What is the major abnormality in Drug C? * (1 Point)

- ◯ Mild reduction in amplification
- ◯ Complete failure of aggregation
- ◯ Delayed coagulation only
- ◯ Increased aggregation

16

Q2. Which pathway is MOST likely affected? * (1 Point)

- A. GP IIb/IIIa-mediated final aggregation pathway
- B. COX-1 pathway
- C. Factor VII pathway
- D. Intrinsic coagulation pathway

17

Q3. Which drug class is MOST likely represented? * (1 Point)

- A. Aspirin-like drug
- B. P2Y12 antagonist
- C. GP IIb/IIIa antagonist
- D. Warfarin-like drug

18

Q4. Confidence score *

- ◯ 1 = Not confident
- ◯ 2 = Slightly confident
- ◯ 3 = Moderately confident
- ◯ 4 = Confident
- ◯ 5 = Very confident

19

Q5. Did AI help your reasoning? *

- ◯ Yes
- ◯ No

SECTION B: Coagulation Cases
Coagulation Case 1 – Drug D

sample control - PT (sec) 12 - aPTT (sec) 32 //// Sample Drug D - PT (sec) 13 - aPTT (sec) 92

20

Q1. Which laboratory test is MOST affected? * (1 Point)

- PT only
- aPTT predominantly
- Platelet aggregation only
- Neither PT nor aPTT

21

Q2. Which pathway is MOST likely affected? * (1 Point)

- A. Intrinsic/common pathway
- B. Extrinsic pathway only
- C. Platelet adhesion pathway
- D. Fibrinolytic pathway

22

Q3. Which drug class is MOST likely represented? * (1 Point)

- A. Heparin-like
- B. Warfarin-like
- C. Direct Xa inhibitor
- D. Aspirin-like

23

Q4. Confidence score *

- 1 = Not confident
- 2 = Slightly confident
- 3 = Moderately confident
- 4 = Confident
- 5 = Very confident

24

Q5. Did AI help your reasoning? *

◯ Yes

◯ No

SECTION B: Coagulation Cases
Coagulation Case 2 – Drug E

sample control - PT (sec) 12 - aPTT (sec) 32 //// Sample Drug E - PT (sec) 34 - aPTT (sec) 40

25

Q1. Which laboratory test is MOST affected? * (1 Point)

- A. PT predominantly
- B. aPTT predominantly
- C. Platelet aggregation only
- D. Neither PT nor aPTT

26

Q2. Which pathway is MOST likely affected? * (1 Point)

- A. Extrinsic pathway
- B. Platelet amplification pathway
- C. GP IIb/IIIa pathway
- D. Fibrinolytic pathway

27

Q3. Which drug class is MOST likely represented? * (1 Point)

- A. Vitamin K–dependent coagulation inhibitor
- B. Aspirin-like drug
- C. Heparin-like drug
- D. GP IIb/IIIa antagonist

28

Q4. Confidence score *

- 1 = Not confident
- 2 = Slightly confident
- 3 = Moderately confident
- 4 = Confident
- 5 = Very confident

29

Q5. Did AI help your reasoning? *

- ◯ Yes
- ◯ No

SECTION B: Coagulation Cases
Coagulation Case 3 – Drug F

sample control - PT (sec) 12 - aPTT (sec) 32 //// Sample Drug F - PT (sec) 20 - aPTT (sec) 45

30

Q1. Which laboratory test is MOST affected? * (1 Point)

- A. PT only
- B. aPTT predominantly
- C. Platelet aggregation only
- D. Both PT and aPTT mildly affected

31

Q2. Which pathway is MOST likely affected? * (1 Point)

- A. Common coagulation pathway
- B. Extrinsic pathway only
- C. Platelet aggregation pathway
- D. Fibrinolysis pathway

32

Q3. Which drug class is MOST likely represented? * (1 Point)

- A. Direct Xa inhibitor
- B. Aspirin-like drug
- C. GP IIb/IIIa antagonist
- D. Vitamin K supplement

33

Q4. Confidence score *

- 1 = Not confident
- 2 = Slightly confident
- 3 = Moderately confident
- 4 = Confident
- 5 = Very confident

34

Q5. Did AI help your reasoning? *

- ◯ Yes
- ◯ No

35

SECTION C: Integrated Mechanistic Reasoning Case Integrated Case – Drug G

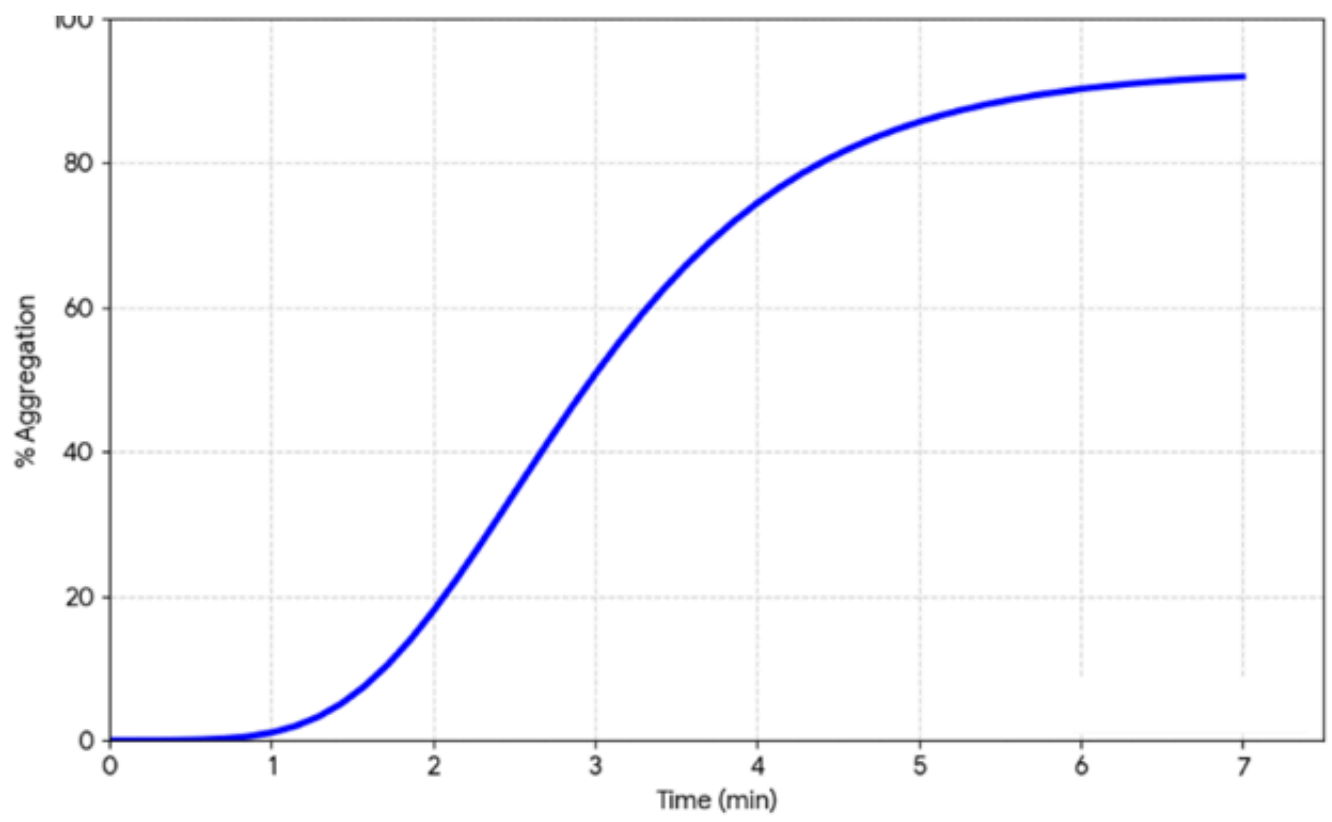

sample control - PT (sec) 12 - aPTT (sec) 32 ////
Sample Drug G - PT (sec) 36 - aPTT (sec) 44

platelet aggregation pattern appears preserved

· initial aggregation response is present

· sustained aggregation remains maintained

· no obvious platelet aggregation abnormality is observed

· coagulation test abnormalities should be interpreted alongside this finding

Q1. Which hemostatic system is primarily affected? *

- A. Vitamin K–dependent coagulation pathway
- B. Platelet aggregation pathway
- C. Fibrinolysis pathway
- D. Intrinsic platelet amplification pathway

37

Q2. Which experimental finding most strongly supports this interpretation? * (1 Point)

- B. Preserved platelet aggregation curve
- C. Absence of fibrinolysis
- A. Marked PT prolongation with milder aPTT prolongation
- D. Delayed platelet lag phase

38

Q3. Which drug class is MOST likely represented? * (1 Point)

- A. Aspirin-like drug
- B. Warfarin-like drug
- C. GP IIb/IIIa antagonist
- D. P2Y12 antagonist

39

Q4. Confidence score *

- ○ 1 = Not confident
- ○ 2 = Slightly confident
- ○ 3 = Moderately confident
- ○ 4 = Confident
- ○ 5 = Very confident

40

Q5. Did AI help your reasoning? *

- ○ Yes
- ○ No

End of Worksheet

This content is neither created nor endorsed by Microsoft. The data you submit will be sent to the form owner.

Microsoft Forms
