# Supplementary material for: Evaluation of a multi-component AI-guided mechanistic reasoning practical in experimental pharmacology: a single-group pre-post study
Source: Front Pharmacol. 2026 Sep 21;17:1937176. doi: 10.3389/fphar.2026.1937176 (PMC13634817; doi:10.3389/fphar.2026.1937176)
Supplement: Supplementary file 4 [file DataSheet4.pdf]

Step 4: Post-Intervention Assessment

Student Instructions
Please complete this assessment independently.

- Do not use AI tools
- Do not use notes or external resources
- Enter your anonymous study ID
- Estimated time: 15–20 minutes

This assessment is intended to evaluate your independent learning after the practical session. Please complete it without using AI tools, notes, internet resources, or assistance from others.

* Required

A. Platelet Pharmacology Knowledge

5 items

3. **Q1. Which platelet receptor is the main pharmacological target of clopidogrel- like drugs?** * (1 Point)

- ◯ P2Y12 receptor
- ◯ GP IIb/IIIa receptor
- ◯ COX-1 enzyme
- ◯ Factor Xa

4. **Q2. Aspirin reduces platelet aggregation mainly by decreasing the production of:** * (1 Point)

- ◯ B. Fibrinogen
- ◯ C. Factor VII
- ◯ D. Antithrombin
- ◯ A. Thromboxane A_2

5. **Q3. Final platelet aggregation depends mainly on:** * (1 Point)

- ◯ B. Vitamin K recycling
- ◯ C. Intrinsic pathway activation
- ◯ A. Fibrinogen binding between activated GP IIb/IIIa receptors
- ◯ D. Factor Xa inhibition

6. **Q4. A platelet-rich plasma sample becomes clearer after agonist exposure. What does this indicate about platelet behavior?** * (1 Point)

- ◯ A. Increased platelet aggregation
- ◯ B. Reduced platelet aggregation
- ◯ C. Increased coagulation factor activity
- ◯ D. Reduced fibrinolysis

7. **Q5. A drug that reduces the secondary wave of platelet aggregation most likely interferes with:** * (1 Point)

- ◯ B. Fibrin degradation
- ◯ A. Platelet amplification signaling
- ◯ C. Red blood cell production
- ◯ D. Prothrombin time measurement

B. Coagulation Pharmacology Knowledge

5 items

8. **Q6. A prolonged PT most strongly suggests impairment of the:** * (1 Point)

- ◯ B. Platelet aggregation pathway
- ◯ C. Thromboxane pathway
- ◯ D. Fibrinolytic pathway
- ◯ A. Extrinsic/common coagulation pathway

9. **Q7. A prolonged aPTT most strongly reflects impairment of the:** * (1 Point)

- ◯ A. Intrinsic/common coagulation pathway
- ◯ B. Platelet adhesion pathway
- ◯ C. COX-1 pathway
- ◯ D. P2Y12 pathway

10. **Q8. A drug reduces synthesis of factors II, VII, IX, and X. Which anticoagulant class best fits?** * (1 Point)

- ◯ B. Platelet ADP receptors
- ◯ A. Vitamin K–dependent clotting factors
- ◯ C. GP IIb/IIIa receptors
- ◯ D. Cyclooxygenase-1

11. **Q9. A sample shows rapid anticoagulation with marked aPTT prolongation mediated through endogenous inhibitor enhancement. Which mechanism fits?** * (1 Point)

- ◯ B. Thromboxane synthesis
- ◯ C. Vitamin K recycling
- ◯ D. Platelet fibrinogen binding
- ◯ A. Antithrombin activity

12. **Q10. A drug prolongs both PT and aPTT mildly while platelet aggregation remains normal. Which coagulation target best explains this?** * (1 Point)

- ◯ B. The platelet amplification pathway
- ◯ C. The COX-1 pathway
- ◯ A. The common coagulation pathway
- ◯ D. The fibrinolytic pathway

C. Experimental Reasoning

10 items

13. **Q11. A platelet aggregation curve shows normal initial aggregation but poor sustained aggregation after ADP stimulation. The most likely mechanism is:** * (1 Point)

- A. P2Y12 inhibition
- B. Factor Xa inhibition
- C. Vitamin K antagonism
- D. Antithrombin deficiency

14. **Q12. A platelet sample shows almost flat aggregation curves after stimulation with ADP, collagen, and thrombin. The most likely mechanism is:** * (1 Point)

- B. COX-1 inhibition
- A. GP IIb/IIIa inhibition
- C. Warfarin-like effect
- D. Direct Xa inhibition

15. **Q13. A platelet sample shows impaired response to an agonist dependent on thromboxane generation, while direct thrombin-induced aggregation remains intact. which pathway is affected?** * (1 Point)

- B. GP IIb/IIIa activation
- A. COX-1/thromboxane pathway inhibition
- C. Factor VII depletion
- D. Direct thrombin inhibition

16. **Q14. A sample has PT 38 seconds and aPTT 42 seconds. The most likely drug effect is:** * (1 Point)

- A. Warfarin-like vitamin K antagonism
- B. Aspirin-like COX inhibition
- C. P2Y12 receptor antagonism
- D. GP IIb/IIIa inhibition

17. **Q15. A sample has PT 13 seconds and aPTT 95 seconds. The most likely drug effect is:** * (1 Point)

- ◯ B. Aspirin-like antiplatelet effect
- ◯ C. Warfarin-like anticoagulation
- ◯ A. Heparin-like anticoagulation
- ◯ D. GP IIb/IIIa inhibition

18. **Q16. A sample has mildly prolonged PT and mildly prolonged aPTT. Platelet aggregation is normal. The most likely mechanism is:** * (1 Point)

- ◯ B. P2Y12 inhibition
- ◯ A. Direct factor Xa inhibition
- ◯ C. COX-1 inhibition
- ◯ D. GP IIb/IIIa inhibition

19. **Q17. A platelet curve shows preserved primary wave but reduced secondary wave. PT and aPTT are normal. Which system is primarily affected?** * (1 Point)

- ◯ B. Coagulation cascade
- ◯ C. Fibrinolysis
- ◯ D. Red blood cell production
- ◯ A. Platelet function

20. **Q18. A simulated sample shows normal platelet aggregation but markedly prolonged PT. Which interpretation is most appropriate?** * (1 Point)

- ◯ A. Coagulation pathway abnormality
- ◯ B. Platelet final common pathway blockade
- ◯ C. Platelet COX-1 inhibition
- ◯ D. P2Y12 receptor blockade

21. **Q19. A drug causes selective prolongation of aPTT with preserved platelet aggregation. Which reasoning approach best identifies the likely mechanism?** * (1 Point)

- ◯ A. Identify affected hemostatic system first, then infer pathway
- ◯ B. Focus only on platelet morphology
- ◯ C. Ignore coagulation results
- ◯ D. Guess based on drug familiarity

22. **Q20. A drug causes reduced sustained aggregation after ADP stimulation but does not prolong PT or aPTT. The most likely drug class is:** * (1 Point)

- ◯ B. Warfarin-like anticoagulant
- ◯ A. P2Y12 antagonist
- ◯ C. Direct Xa inhibitor
- ◯ D. Heparin-like anticoagulant

End of Post test

This content is neither created nor endorsed by Microsoft. The data you submit will be sent to the form owner.

Microsoft Forms
