# Supplementary material for: Evaluation of a multi-component AI-guided mechanistic reasoning practical in experimental pharmacology: a single-group pre-post study
Source: Front Pharmacol. 2026 Sep 21;17:1937176. doi: 10.3389/fphar.2026.1937176 (PMC13634817; doi:10.3389/fphar.2026.1937176)
Supplement: Supplementary file 5 [file DataSheet5.pdf]

Step 5: Perception and Acceptability Survey

* Required

AI-supported learning perception

1. Anonymous Study ID

2. The AI-guided practical helped me understand antiplatelet drug mechanisms. *

- Strongly agree
- Agree
- Neutral
- Disagree
- Strongly disagree

3. The AI-guided practical helped me understand anticoagulant drug mechanisms. *

- Strongly agree
- Agree
- Neutral
- Disagree
- Strongly disagree

4. The AI-guided practical helped me connect drug mechanisms to experimental readouts. *

- ◯ Strongly agree
- ◯ Agree
- ◯ Neutral
- ◯ Disagree
- ◯ Strongly disagree

5. The AI-guided practical helped me interpret platelet aggregation curves. *

- ◯ Strongly agree
- ◯ Agree
- ◯ Neutral
- ◯ Disagree
- ◯ Strongly disagree

6. The AI-guided practical helped me interpret PT and aPTT results. *

- ◯ Strongly agree
- ◯ Agree
- ◯ Neutral
- ◯ Disagree
- ◯ Strongly disagree

Experimental reasoning

7. The AI-guided practical improved my ability to reason from data to mechanism. *

- ◯ Strongly agree
- ◯ Agree
- ◯ Neutral
- ◯ Disagree
- ◯ Strongly disagree

8. The AI-guided practical helped me identify which pathway was affected before selecting the drug class. *

- ◯ Strongly agree
- ◯ Agree
- ◯ Neutral
- ◯ Disagree
- ◯ Strongly disagree

9. The AI-guided practical made me more confident in interpreting simulated pharmacology data. *

- ◯ Strongly agree
- ◯ Agree
- ◯ Neutral
- ◯ Disagree
- ◯ Strongly disagree

AI acceptability

10. The AI guidance was useful without simply giving me the answer. *

- ◯ Strongly agree
- ◯ Agree
- ◯ Neutral
- ◯ Disagree
- ◯ Strongly disagree

11. I would support using AI-guided reasoning activities in future pharmacology practicals. *

- ◯ Strongly agree
- ◯ Agree
- ◯ Neutral
- ◯ Disagree
- ◯ Strongly disagree

Safety/limitations

12. The AI sometimes made the reasoning process more confusing. *

- Strongly agree
- Agree
- Neutral
- Disagree
- Strongly disagree

13. I felt I needed instructor guidance in addition to AI support. *

- Strongly agree
- Agree
- Neutral
- Disagree
- Strongly disagree

This content is neither created nor endorsed by Microsoft. The data you submit will be sent to the form owner.

Microsoft Forms
